# Magnesium–Phenolic Nanoeditor Refining Gliomatous T Cells for Metalloimmunotherapy

**DOI:** 10.1021/acsnano.4c13388

**Published:** 2024-12-19

**Authors:** Wenxi Li, Hao Tian, Ziliang Yan, Xinying Yu, Bei Li, Yunlu Dai

**Affiliations:** † Cancer Centre and Institute of Translational Medicine, Faculty of Health Sciences, University of Macau, Macau SAR 999078, China; ‡ MoE Frontiers Science Center for Precision Oncology, University of Macau, Macau SAR 999078, China

**Keywords:** magnesium-phenolic coordination, glioblastoma, metalloimmunotherapy, macrophage polarization, cytotoxic T lymphocytemodulation

## Abstract

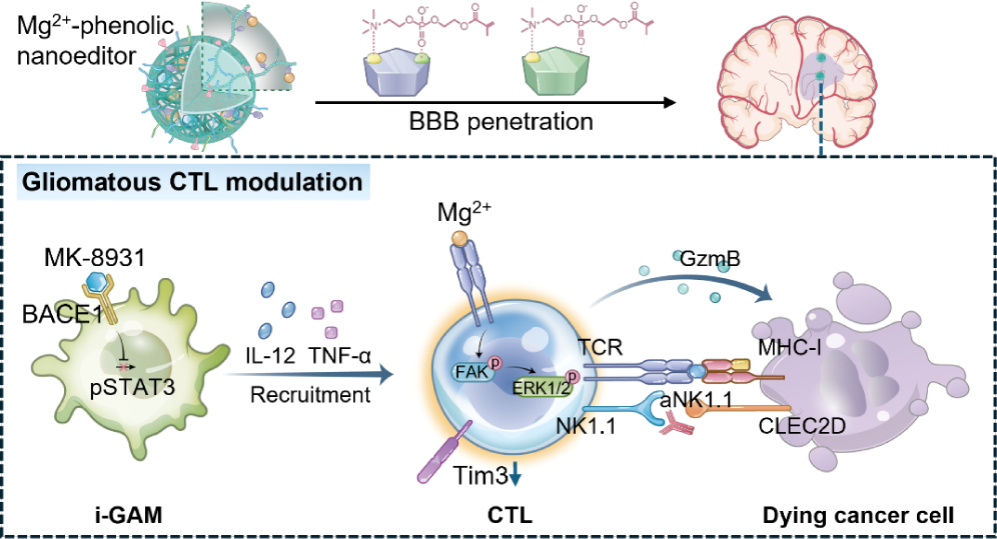

More than the sparse infiltration in glioblastoma, cytotoxic T lymphocytes (CTLs) also function inefficiently and overexpress the inhibitory markers, especially the identified NK cell receptor (NK1.1). However, most studies solely focus on how to augment tumor-infiltrating CTLs and overlook their killing maintenance. Metalloimmunotherapy has been proven to improve the functionalities of CTLs, but it has barely adapted to glioblastoma due to the severe limitations of safe delivery and the brain’s physiological properties. Herein, we synthesized an amphipathic polyethylene glycol (PEG) polymer (designated as MPP) modified with the choline analogue 2-methacryloyloxyethyl phosphorylcholine (MPC) and polyphenol moieties to customize a nanoeditor (Mg^2+^@MK-8931@MPP) by coordinating Mg^2+^ and entrapping the hydrophobic BACE1 inhibitor MK-8931, then precisely redressing the gliomatous CTL sparsity and cytotoxic dysfunction. Upon MPC-assisted local accumulation in glioblastoma, Mg^2+^@MK-8931@MPP nanoeditors release MK-8931 to repolarize M2-like macrophages, facilitating CTL infiltration quantitatively. The cenogenetic immune adjuvant Mg^2+^ ulteriorly fortifies the T-cell receptor downstream signals to enhance the functionality of the ingoing CTLs in quality, leading to the secretion of high-level antitumor cytokines and cytotoxic proteins. Further blocking the inhibitory NK1.1 on CTLs by anti-NK1.1 antibodies can extend their cytolytic endgame. Studies on T-cell-deficient and wild-type mouse models support the immunomodulating feasibility of Mg^2+^@MK-8931@MPP. This gliomatous CTL-tailored strategy concurrently broadens metalloimmunotherapy to glioblastoma treatment and highlights the necessity of enforcing gliomatous CTLs’ functionality.

## Introduction

Glioblastoma multiforme (GBM), an aggressive and malignant grade IV brain tumor arising from astrocytes in the central nervous system, encounters poor clinical response to burgeoning immunotherapy.^1,2^ This intractability is highly associated with an intricate glioblastoma microenvironment (GME)·^3^ Specifically, at least 30% of the GME is occupied by the suppressive glioblastoma-associated macrophages (s-GAMs, M2-like phenotype) to support glioblastoma growth and remote invasion.^4,5^ These s-GAMs promote the generation of regulatory T cells (T_reg_)^6^ but curb the gliomatous infiltration of cytotoxic T lymphocytes (CTLs), contributing to a CTL-sparse GME.^7^ Repolarizing s-GAMs to a glioblastoma-inhibitory phenotype (i-GAMs, M1-like phenotype) was once considered a fundamental strategy to tackle the GME immune resistance.^8^ For example, β-site amyloid precursor protein-cleaving enzyme 1/2 (BACE1/2) inhibitor verubecestat (MK-8931), a retired Alzheimer’s disease (AD) drug, was repurposed to macrophage repolarization against GBM.^9^ However, glioblastoma seizes the organism’s immune tolerance behavior to induce low T-cell receptor (TCR) affinity and high immunoinhibitory marker expression for CTL unresponsiveness.^10−13^ Neglecting the persistence of the CTL effector function but only improving CTL infiltration is incompetent for GBM therapy.^14−16^ The key to problem-solving lies in the functionality of gliomatous CTLs.

Metalloimmunology displays powerful capabilities for modulating T cells against cancer, such as the well-known manganese ions (Mn^2+^), sensitizing the cGAS-STING pathway to trigger T-cell activation.^17−19^ However, most metal ions (*e.g.*, widely studied metal ions Mn^2+^, Zn^2+^, and Cu^2+^) usually induce neurodegenerative diseases due to the complex physiological characteristics of the brain, thus confining the application of metalloimmunology in glioblastoma treatment.^20,21^ Poles apart from other immunomodulatory metal ions, magnesium ions (Mg^2+^) supplementation substantially benefits neuroprotection and brain health.^22^ More importantly, Mg^2+^ is determined to reshape T-cell effector function via stimulating the T-cell receptor (TCR), its downstream focal adhesion kinase (FAK), and extracellular signal-regulated protein kinases 1 and 2 (ERK1/2) signals.^23^ Employing Mg^2+^ as a cenogenetic immune adjuvant to fortify the functionality of the ingoing CTLs recruited by M1-like i-GAMs is expected to redress gliomatous CTL sparsity and cytotoxic dysfunction concurrently.

Meanwhile, the NK cell receptor (NK1.1) is a newly identified immunoinhibitory marker that is hyperexpressed on the glioblastoma-infiltrating T cells and is readily bound by the CLEC2D ligand on glioblastoma cells, ultimately hampering CTL cytolytic killing and cytokine secretion.^24,25^ These qualitative dysfunctions also contribute to low abundance of activated markers (CD69, CD25), degranulated markers (CD107a, Granzyme B), and functional markers (IFN-γ, TNF-α), *etc*.^26^ Given that, collaborating macrophage repolarization drug MK-8931 and the immune adjuvant Mg^2+^ ions with anti-NK1.1 antibodies (aNK1.1) may specifically negotiate the deficiencies of gliomatous CTL infiltration, cytotoxicity, and serial killing capability. Moreover, considering the hydrophobicity of MK-8931 and the systemic effect of Mg^2+^, a biocompatible and applicable carrier should be provided to rescue the scarcity and dysfunction of gliomatous CTLs in a precise and safe delivery manner.

**Scheme 1:**
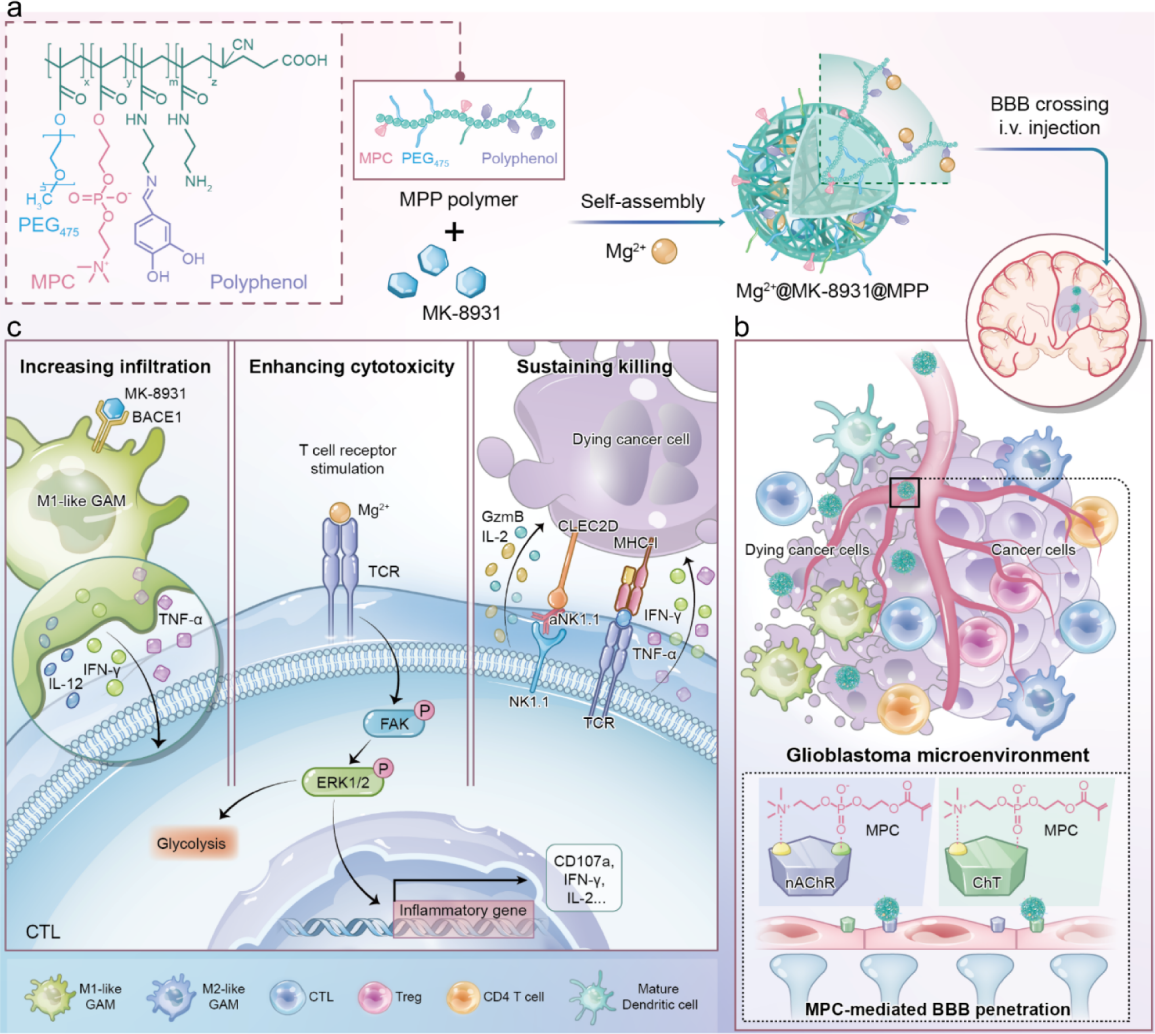
(a) Schematic Diagram of Mg^2+^@MK-8931@MPP Nanoeditor Self-Assembly Process *via* Coordinating Mg^2+^ with Polyphenol-Enriched MPP and Hydrophobically Entrapping MK-8931 Inhibitor for Precise Glioblastoma Metalloimmunotherapy. (b) I.v. Injected Mg^2+^@MK-8931@MPP Nanoeditor Interacts with the nAChRs and ChTs Receptors by MPC Group for Efficient BBB Penetration and Glioblastoma Accumulation. (c) Mechanism Diagram of Mg^2+^@MK-8931@MPP Nanoeditor Cooperating with aNK1.1 for Gliomatous CTL Regulation: 1) MK-8931 Repolarizes s-GAMs into i-GAMs for Promoting CTL Infiltration. 2) Mg^2+^ ions Remodel the TCR Signals of CTLs for Enhancing CTL Cytotoxicity. 3) The Additional Aidance of aNK1.1 Blocks the Immunoinhibitory NK1.1–CLED2C Interaction for Persisting CTL Serial Killing Capacity.

Hence, we synthesized an ingenious amphiphilic polyethylene glycol (PEG) polymer that was consisted of the choline analogue 2-methacryloyloxyethyl phosphorylcholine (MPC) and pyrocatechol moieties through stepwise reversible addition–fragmentation chain transfer polymerization, designated as MPC-PEG-Polyphenol (MPP for short) polymer. The polyphenol units and amphiphilic properties of MPP enabled its coordination with Mg^2+^ and hydrophobically encapsulated the BACE1 inhibitor (MK-8931), forming a self-assembled Mg^2+^@MK-8931@MPP nanoeditor (Scheme 1). After intravenous injection (i.v.) of Mg^2+^@MK-8931@MPP, MPC units promoted blood-brain barrier (BBB) penetration for sufficient intraglioblastoma accumulation by interacting with nicotinic acetylcholine receptors (nAChRs) and choline transporters (ChTs).^27,28^ Next, pH-responsive release of MK-8931 repolarized s-GAMs into i-GAMs *via* activating trans-interleukin 6 (IL-6)-signal transducer and activator of transcription 3 (STAT3) signaling,^9^ thereby promoting CTL infiltration into the glioblastoma niche. Regarding CTL functionality, the immune adjuvant Mg^2+^ remodeled the TCR signaling of glioblastoma-infiltrating CTLs to potentiate their cytotoxic killing capacity.^23^ Moreover, the cytotoxicity of Mg^2+^-regulated CTLs could be further persisted by additional aNK1.1 blocking.^25^ In the end, two mouse models, including T-cell-lacking Rag^–/–^ and wild-type C57BL/6J, were leveraged to demonstrate the glioblastoma-killing performance of CTLs optimized by Mg^2+^@MK-8931@MPP.^29^ Such a nanoeditor may broaden the horizon of glioblastoma treatment to precise metalloimmunotherapy, especially for quantity- and quality-oriented regulation of CTL potency.

## Results and Discussion

### Nanoeditor Characterizations and Mg^2+^–Polyphenol Coordination Effect

**Figure 1:**
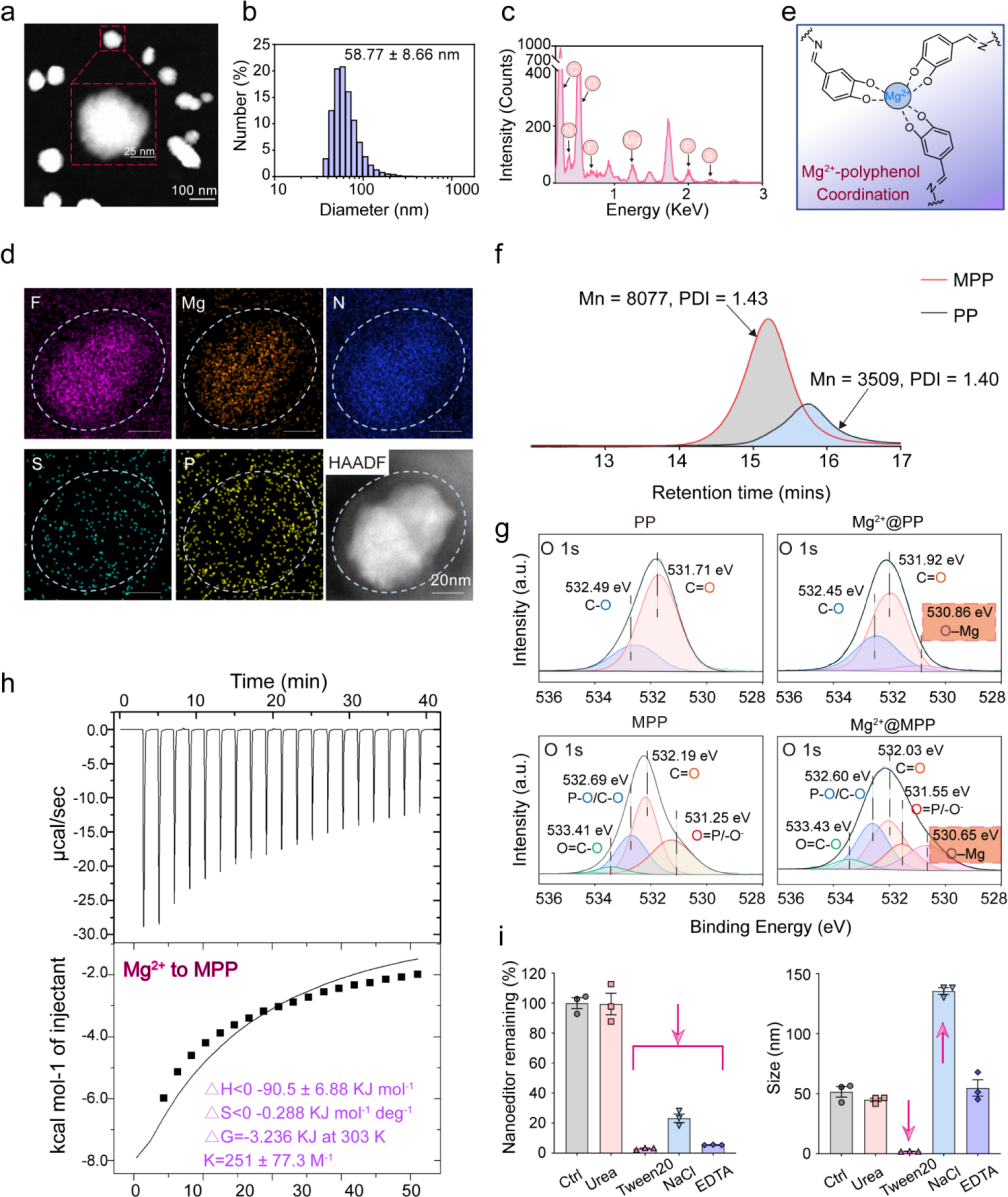
Characterizations of Mg^2+^@MK-8931@MPP nanoeditors. (a) Representative HAADF-STEM image with an enlarged area. (b) Dynamic light scattering measurement. (c,d) EDX spectrum (c) and representative energy dispersive X-ray (EDX) mapping images and HAADF-STEM image (d) of Mg^2+^@MK-8931@MPP nanoeditor. (e) Schematic diagram of the Mg^2+^-polyphenol coordination mechanism. (f) GPC measurements of MPP and PP polymers. (g) Fit curves and assigned photoelectron peaks (O 1s) in X-ray photoelectron spectroscopy (XPS) spectra. (h) Thermodynamic ITC curve obtained by titration of MgCl_2_ in MPP solution at 29.85 °C (means *T* = 303 K). The top panels represent the raw data, and the bottom panels represent the fitted curves of molar heat changes upon different MPP-to-MgCl_2_ molar ratios. (i) Concentration and size variations of Mg^2+^@MK-8931@MPP (at a concentration of 4 mg MPP) upon the interaction-dependent disassociation in 4 M Urea, 100 mM Tween20, 2 M NaCl, and 150 mM EDTA after 12 h coincubation, respectively.

The MPP polymer was synthesized by routes shown in Figure S1 and characterized by proton nuclear magnetic resonance (^1^H NMR) step by step Figures S2–S7.^30^ Nanoeditors with diverse weight ratios (Mg^2+^: MK-8931: MPP) were then synthesized by ultrasonication to evaluate the size distribution and particle stability for formula optimization. The nanoeditor with a weight ratio of 6:3:20 showed negligible size changes within 7 days (Figure S8). The spherical morphology of the nanoeditors (hereafter termed the ratio 6:3:20) was observed using high-angle annular dark-field scanning transmission electron microscopy (HAADF-STEM) (Figure 1a), with a hydrodynamic diameter of 58.77 ± 8.66 nm (Figure 1b). Energy-dispersive X-ray spectroscopy (EDX) elemental mapping and spectra analysis identified the homogeneous distribution of fluorine (F), magnesium (Mg), phosphorus (P), sulfur (S), and nitrogen (N) in Mg^2+^@MK-8931@MPP, confirming the successful encapsulation of Mg^2+^ ions and MK-8931 (Figure 1c,d). The rapid self-assembly of Mg^2+^@MK-8931@MPP nanoeditor suggested the Mg^2+^-phenolic coordination and hydrophobic encapsulation of MK-8931 (Figure 1e).^31,32^ To better explore the nanoformation mechanism and MPC moiety function, we synthesized a PEG-polyphenol polymer without MPC modification (denoted as PP) as a control polymer and conducted a series of verifications. The molecular weights and weight distribution analyses of MPP and PP polymers were determined by gel permeation chromatography (GPC) (Figure 1f).

X-ray photoelectron spectroscopy (XPS) was subsequently used to substantiate the coordination effect between Mg^2+^ and the MPP polymer by analyzing the O 1s and Mg 1s spectra. Compared with PP, we identified a new photoelectron peak of the O 1s at the 530.86 eV binding energy position in Mg^2+^@PP nanoparticles (PP interacted with Mg^2+^) (Figure 1g). The same peak was observed in the Mg^2+^@MPP group at 530.65 eV but not in the MPP group, indicating a newly formed O–Mg bond from the binding between Mg^2+^ ions and phenolic hydroxyl groups.^33−35^ This binding was also supported by the peak shift to ∼1303 eV in the Mg 1s spectra of Mg^2+^@PP and Mg^2+^@MPP groups compared to 1304.86 eV in MgCl_2_ (Figure S9).^36,37^ We also employed isothermal titration calorimetry (ITC) to analyze the thermodynamic change upon the interaction between MPP and Mg^2+^ for double corroboration. As shown in Figures 1h and S10, the enthalpy change predominated the coordination effect between polyphenol-enriched MPP and MgCl_2_, which was driven by a high Δ*H* of −90.5 ± 6.88 kcal mol^–1^, despite a faintly unfavorable entropic contribution (−*T*Δ*S*= 0.087 kcal mol^–1^), the negative Gibbs free energy change Δ*G* (−3.236 KJ, Δ*G* = Δ*H*–*T*Δ*S*, *T* is kelvin temperature) still revealed the interaction was spontaneous. Similar strongly spontaneous interaction was observed in PP-to-MgCl_2_ (Δ*G* = −3.58 KJ), but weak interaction occurred in MPC-to-MgCl_2_ (Δ*G* = −2.286 KJ), supporting the Mg^2+^–polyphenol interaction.^38^ Meanwhile, there is more than a 5-fold affinity between the polymer and MgCl_2_ in MPP-to-MgCl_2_ (*K* = 251 ± 77.3 M^–1^) and PP-to-MgCl_2_ (*K* = 222 ± 10.3 M^–1^) compared to MPC-to-MgCl_2_ (*K* = 44.4 ± 19.4 M^–1^), which proves the credibility of the Mg^2+^–polyphenol interaction. Moreover, the stretching vibration at 578 cm^–1^ of the Mg^2+^@PP group in the Fourier transform infrared spectroscopy (FTIR) spectrum reaffirmed the Mg^2+^–phenolic coordination effect (Figure S11).^39^ To better elucidate the self-assembly mechanism of nanoeditors, Nanoparticle Tracking Analysis (NTA) combined with Dynamic light scattering (DLS) was embodied to measure the concentration and size variations with the action of different solutions (Figure 1i). Almost all of the Mg^2+^@MK-8931@MPP nanoeditor (at a concentration of 4 mg MPP) was decomposed in 100 mM Tween20 and about 80% in 150 mM ethylenediaminetetraacetic acid (EDTA). At the same time, urea did not induce any changes, indicating that the hydrophobic effect and coordination effect dominated the nanoeditor assembly, irrespective of hydrogen bonding. Meanwhile, the presence of NaCl resulted in a 3-time negative correlation between size increase and concentration decrease, indicating that electrostatic interactions contributed to the stability of the nanoeditor.

Next, the coordination rate and loading capacity of Mg^2+^ in Mg^2+^@MK-8931@MPP nanoeditors were characterized by inductively coupled plasma mass spectrometry (ICP-MS) and quantitated to be 30.86 ± 1.41% and 7.47 ± 0.32%, respectively. Meanwhile, ultraviolet–visible (UV–vis) spectroscopy was applied to examine the encapsulation efficiency and loading capacity of MK-8931 in Mg^2+^@MK-8931@MPP, which was quantified to be 92.1% and 9.03%, respectively (Figure S12). Since metal-phenolic coordination is pH-dependent,^40^ MK-8931 and Mg^2+^ were proven to be gradually released from the Mg^2+^@MK-8931@MPP in acidic buffer (pH 6.5), approximately 50% of MK-8931 and 32.66% of Mg^2+^ within 24 h (Figure S13). Hereto, the fabricated Mg^2+^@MK-8931@MPP has been comprehensively characterized, which exhibits an acidity-responsive property for drug release.

### BBB Penetrability of Mg^2+^@MK-8931@MPP Nanoeditor

**Figure 2:**
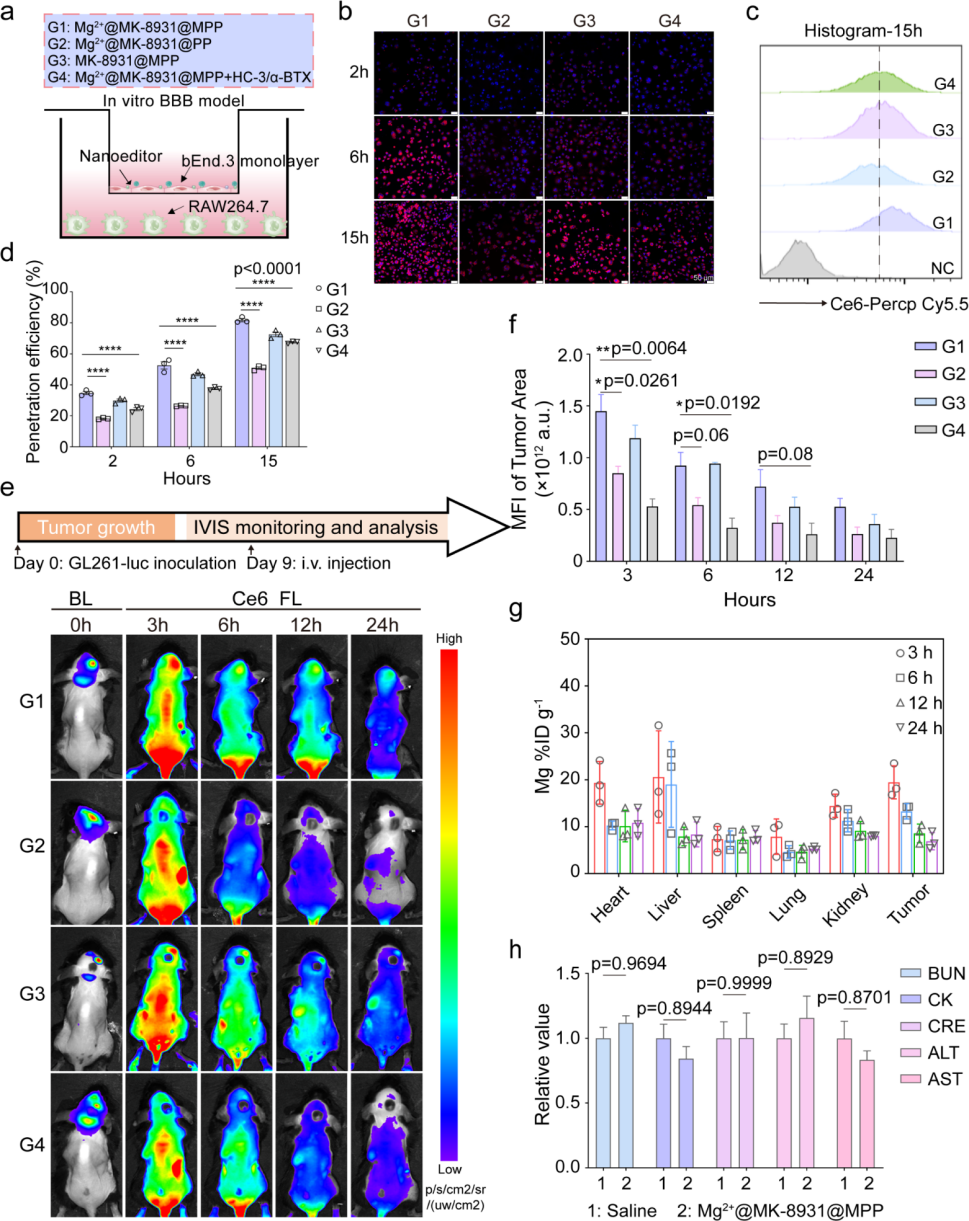
BBB penetration capability evaluation of Mg^2+^@MK-8931@MPP nanoeditor. (a) Graphical illustration of the *in vitro* BBB model. (b) Representative CLSM images of Mg^2+^@MK-8931@MPP (G1), Mg^2+^@MK-8931@PP (G2), MK-8931@MPP (G3), and Mg^2+^@MK-8931@MPP+HC-3/α-BTX (G4) penetrating from the upper chamber (b. End3 cells, endothelial monolayer) into the bottom chamber (RAW264.7 cells, bottom uptake layer) under 2, 6, and 15 h coincubation. (c,d) Representative flow cytometric histograms and flow cytometric quantification of penetrated nanoparticles in the bottom chamber under 2, 6, and 15 h coincubation. (e) Representative biodistribution images of diverse groups after i.v. injection into GL261-bearing C57BL/B mice. (f) Fluorescence quantification of nanoparticle accumulation at glioblastoma sites at different time points. (g) Biodistribution quantitative analyses of Mg^2+^@MK-8931@MPP accumulated in diverse tissues by measuring magnesium concentration. Mg concentrations were normalized as the cumulative dose of Mg per gram of the corresponding tissue as a percentage of the injected dose (%ID g^–1^). (h) The relative value of biochemical analysis results by comparing the Mg^2+^@MK-8931@MPP nanonetwork group with the saline group *in vivo*. *n* = 3 mice at each time point per group in panels d, f, g. *n* = 5 mice per group in panel h. Statistical significance was calculated *via* two-way ANOVA with Tukey’s multiple comparisons in panel d, two-tailed unpaired *t* test in panel f, and two-way ANOVA with Sidak’s multiple comparisons in panel h. *p* values < 0.05 were considered significant: *p < 0.05, **p < 0.01, ***p < 0.001, and ****p < 0.0001.

To confront glioblastoma, one necessary precondition is that Mg^2+^@MK-8931@MPP nanoeditors should efficiently cross the BBB. Three kinds of nanoparticles were fabricated for further investigation, including Mg^2+^@MK-8931@MPP (G1), Mg^2+^@MK-8931@PP (G2), and MK-8931@MPP (G3). All of them were prepared using a Chlorin e6 (Ce6)-conjugated polymer for easy tracking (Figures S14 and S15). Moreover, the irreversible inhibitors, α-bungarotoxin (α-BTX) for nicotinic acetylcholine receptors (nAChRs) and hemicholinium-3 (HC-3) for choline transporters (ChTs), were used to confirm the function of MPC on BBB penetration.^27^ An *in vitro* BBB model was established by seeding the brain-derived endothelial cell line bEnd.3 into the upper chamber and RAW264.7 cells at the bottom to investigate BBB-penetrating behaviors, as detailed in the Methods section (Figure 2a).^41,42^ We added nanoparticles into the upper chamber once the transepithelial/transendothelial electrical resistance (TEER) of bEnd.3 cell layer reached 150–300 Ω·cm^2^, indicating that an integrated monolayers was formed. RAW264.7 cells at the bottom were collected after 2, 6, and 15 h, separately, for confocal laser scanning microscopy (CLSM) imaging and flow cytometry (FCM) analyses. The only difference in G4 group is that α-BTX and HC-3 were replenished to the upper chamber 1 h in advance. As shown in Figure 2b-d, by comparison with the 18.2% ± 0.57% penetration at 2 h and 50.9% ± 1.44% at 15 h of Mg^2+^@MK-8931@PP (G2), faster BBB crossing occurred in Mg^2+^@MK-8931@MPP (G1) upon the assistance of MPC, which exhibited 34.7% ± 1.73% penetration within the initial 2 h and reached up to 81.8% ± 1.82% within 15 h. The apparent BBB-penetrating variances indicated that the MPC linkage endowed Mg^2+^@MK-8931@MPP nanoeditors with superior BBB-penetrating capability, and the significantly decreased penetration rate in Mg^2+^@MK-8931@MPP + HC-3/α-BTX (G4) supports this view. Besides, the penetration of MK-8931@MPP (G3) was a little lower than Mg^2+^@MK-8931@MPP (G1), implying that the positive charge from Mg^2+^ also benefited the BBB penetration of our nanoeditors *via* adsorptive transcytosis.^43^

Subsequently, an orthotopic GL261-bearing mouse model was employed to further examine the MPC function in promoting BBB-penetration efficiency *in vivo*. As measured by the *in vivo* fluorescence imaging system (IVFIS) in Figure 2e,f, Mg^2+^@MK-8931@MPP (G1) nanoparticles rapidly accumulated into the glioblastoma site after intravenous administration, peaking at 3 h post-injection and retaining for at least 24 h despite gradual metabolism. Oppositely, Mg^2+^@MK-8931@PP (G2) showed low intratumoral accumulation and was metabolized mostly within 24 h, demonstrating the excellent BBB-penetration-promoting effect of the MPC moiety. The addition of HC-3 and α-BTX greatly hindered the ability of Mg^2+^@MK-8931@MPP (G4) to cross the BBB. When Mg^2+^ was removed, MK-8931@MPP (G3) also showed a relatively superior accumulation rate that was only slightly weaker than Mg^2+^@MK-8931@MPP (G1) but better than Mg^2+^@MK-8931@PP (G2), declaring the contribution of MPC to penetration behavior. We then harvested the major organs (heart, liver, spleen, lung, and kidney) and brain tumors at diverse time points (3, 6, 12, and 24 h) for biodistribution analyses by ICP-MS measurement. The highest accumulation of Mg %ID per brain tumor gram in the Mg^2+^@MK-8931@MPP group was identified at 3 h postinjection and gradually metabolized from the liver and kidney, demonstrating acceptable penetration efficiency and security controllability (Figure 2g). Both *in vitro* and *in vivo* results expounded the BBB crossing mechanism of Mg^2+^@MK-8931@MPP and highlighted the positive contribution of MPC. We also collected sera on day 7 after nanoeditor injection to further verify the security and feasibility of Mg^2+^@MK-8931@MPP for *in vivo* investigations. The negligible variations of biochemical analysis results indicated the safety-applied potential of these nanoeditors *in vivo*. The measured biochemical parameters included blood urea nitrogen (BUN), creatine kinase (CK), creatinine (CRE), aspartate transaminase (AST), and alanine aminotransferase (ALT) (Figure 2h).

### GAMs Polarization from S-GAMs to I-GAMs

We adopted bone marrow-derived macrophages (BMDMs) to investigate the phenotype-polarizing capacity of the BACE1 inhibitor MK-8931 *in vitro* (all *in vitro* gating strategies are shown in Figure S16), which ascertained that MK-8931 could educate the immunosuppressive BMDMs (CD206^+^ M2-like BMDMs) into immunosupportive ones (CD80^+^ M1-like BMDMs) in a dose-dependent manner (Figure S17a). For coculturing GL261 and pretreated BMDMs (Figure S17b), MK-8931 (50 μg mL^–1^) increased around one-fold phagocytosis of BMDMs to GL261 cells compared to the 17.8 ± 2.7% of naïve BMDM. Assembling MK-8931 into the MPP polymer (MK-8931@MPP) ascended the phagocytosis to 42.2 ± 2.0%, indicating the excellent BMDM-polarizing capacity of MK-8931 and its nanoformation.

**Figure 3:**
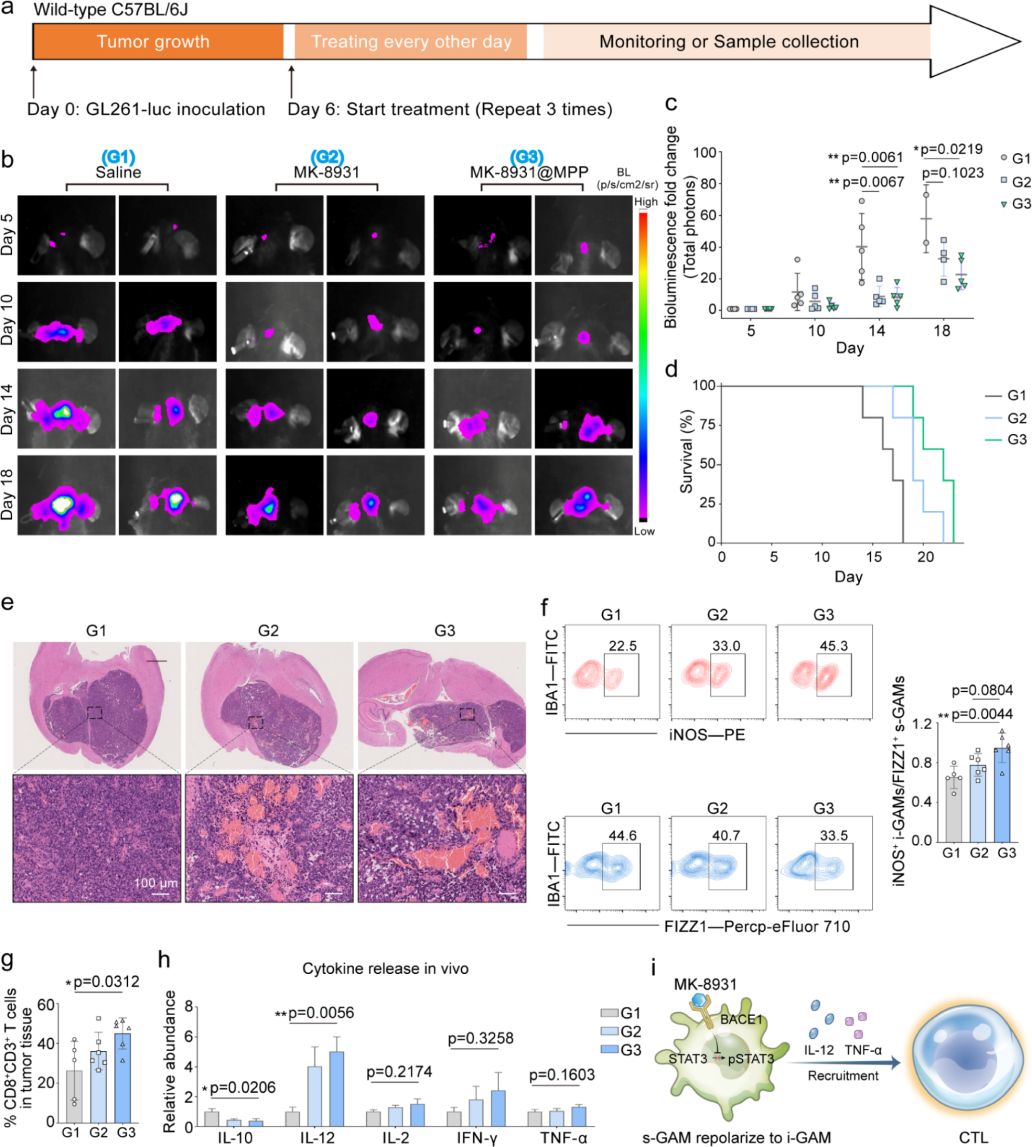
Macrophage polarization capability of MK-8931@MPP. (a,b,c) Schematic illustration (a), representative bioluminescence images (b), and bioluminescence fold change analyses (c) of GL261-luc-bearing mice treated with Saline, MK-8931, and MK-8931@MPP (MK-8931:6.9 mg per kg body weight). (d) Mouse survival per group (*n* = 5 per group). (e) H&E staining of mice brains. f. Representative flow cytometry plots and quantitative analyses of i-GAM/s-GAM ratio under diverse treatments. (g) CD8 T cells ratio in GME (CD8a^+^CD3e^+^ gating on CD3e). (h) Relative abundance of cytokines release profiles in sera. Relative abundances are calculated by dividing each value by the average value of the saline group. (i) Schematic illustration of MK-8931@MPP-induced GAM polarization for CTL recruitment. *n* = 5 in Saline group, *n* = 6 in MK-8931 and MK-8931@MPP groups in panels f-h. All flow data presented here were gated on the upper level, as shown in Figure S19. (f,g) Statistical significance was calculated *via* one-way ANOVA with Tukey’s multiple comparisons. For (h), data are presented as the mean ± standard error of the mean (SEM), and statistical significance was calculated *via* an unpaired two-tailed *t* test with two-group comparisons. *p* values < 0.05 were considered significant: *p < 0.05, **p < 0.01, ***p < 0.001, and ****p < 0.0001.

To examine the *in vivo* GAM-polarizing performance of MK-8931, glioblastoma-bearing (orthotopic GL261) mice were intravenously administered with saline, MK-8931, and MK-8931@MPP nanoparticles, respectively, as Figure 3a scheme depicted. On day 6 post-tumor inoculation, mice bearing glioblastoma were randomized into three groups (5 mice per group) to initiate the therapeutic regimen. GL261 cells were gene-edited with luciferin expression to visualize their intracranial development. As shown in Figure 3b,c, all mice in the saline (G1) group showed strong bioluminescence in the brain tissue within 14 days, indicating rapid tumor growth. In contrast, MK-8931@MPP treatment (G3) decreased brain bioluminescence and barely affected mice weight compared to the control baseline (Figure S18). Further evidence showed that MK-8931@MPP prolonged the 60% lifespan to more than 19 days (Figure 3d) and shrunk the glioblastoma area (Figure 3e), which explicitly inhibited the glioblastoma progression and improved mice survival.

We afterward harvested treated samples (tumor tissues, lymph nodes, sera, and spleen) to phenotype immune cells induced by MK-8931@MPP. The *in vivo* gating strategy was shown in Figure S19. As shown in Figure 3f, the GAMs upon MK-8931@MPP treatment (G3) preferentially differentiated into i-GAM phenotype (iNOS^+^IBA1^+^) instead of s-GAMs (FIZZ1^+^IBA1^+^), with a superior i-GAM/s-GAM ratio of 0.95 ± 0.15 in comparison to 0.65 ± 0.11 in the saline group (G1) or 0.78 ± 0.11 in the MK-8931 group (G2). These i-GAMs have been reported to enable a positive contribution to the antitumor immune response.^44,45^ In the lymph nodes, higher proportions of mature dendritic cells and CD8^+^ T cells were identified after MK-8931@MPP therapy (Figure S20). Likewise, MK-8931@MPP therapy (G3) displayed higher glioblastoma-infiltrating CTLs at a ratio of 45 ± 7.85% versus 26.33 ± 14.73% in the saline group (G1) (Figures 3g and S21). On the contrary, glioblastoma infiltration of regulatory T cells (T_reg_) was considerably inhibited by MK-8931@MPP ascribing to the GAM transformation to a proinflammatory phenotype, which would avail GBM elimination (Figure S22). Further cytokine release results, including decreased anti-inflammatory cytokine (IL-10) and upregulated pro-inflammatory cytokine (IL-12, TNF-α, IL-2, and IFN-γ), reconfirmed the positive modulation of MK-8931@MPP on antitumor immunity (Figures 3h and S23). In a convincible sum-up, the glioblastoma therapeutic effectiveness here is associated with the MK-8931@MPP-induced enhancement of the i-GAM/s-GAM ratio and its serial immune response (Figure 3i).

### Effector Function Enhancement of CTLs

**Figure 4:**
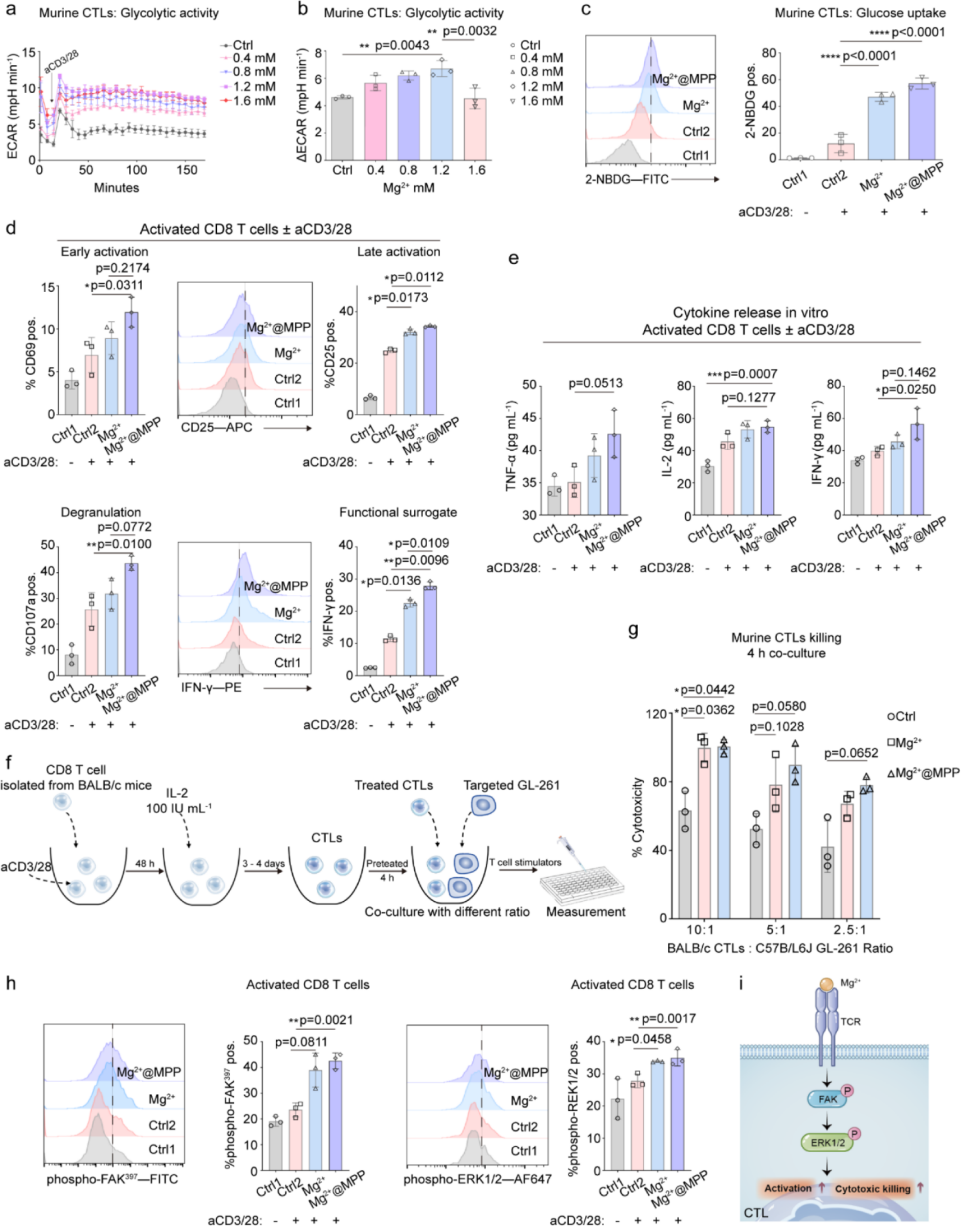
Mg^2+^ ions reshaped T-cell cytotoxicity *in vitro*. Glycolytic activity under Mg^2+^ effect of murine CTLs. Δ*E*CAR was determined by subtracting the baseline from the ECAR maximum to quantify glycolytic activity. (c) Glucose uptake by activated murine CTLs (0.8 mM Mg^2+^). (d) Quantitative analyses of CD69 and CD107a expression on activated CD8 T cells. Representative flow cytometry plots and quantitative analyses of CD25 and IFNγ expression. (e) Corresponding cytokine secretion level in supernatant of activated CD8 T cells. (f) Schematic illustration of the investigation of CTL killing activity. (g) A 4 h killing assay of CTLs was performed using varying ratios of BALB/c CTLs to the target (GL261 cancer cells). (h) Representative histogram and quantitative analyses of the phosphorylation of FAK at the Tyr397 site and the phosphorylation of ERK1/2 at Thr202/Thr204 sites on activated CD8 T cells. (i) Mg^2+^ functions on TCR to sense its downstream pathway by phosphorylating FAK and ERK1/2, then promoting CD8 T-cell activation and cytotoxic killing. *n* = 3 per group. All flow data presented here were gated on the upper level, as shown in Figure S16. For (b-e), statistical significance was calculated *via* one-way ANOVA with Tukey’s multiple comparisons. For (g, h), statistical significance was calculated *via* an unpaired two-tailed *t* test with two-group comparisons. *p* values < 0.05 were considered significant: *p < 0.05, **p < 0.01, ***p < 0.001, and ****p < 0.0001.

As aforementioned, conjugating Mg^2+^ on Mg^2+^@MK-8931@MPP aims to strengthen CTL cytotoxicity for potent metalloimmunotherapy.^23^ We prepared Mg^2+^@MPP nanoparticles by coordinating the MPP with Mg^2+^. The three functional components, including Mg^2+^, MK-8931, and MPP, demonstrated low toxicity toward T-cell viability (Figure S24). Given that the glycolytic activity of CTLs positively correlates with their effector function, CTL glycolysis was evaluated here by measuring the extracellular acidification rate (ECAR).^46^ From Figure 4a,b, we identified that Mg^2+^ markedly raised the ECAR level of CTLs preactivated by anti-CD3/-CD28 antibodies (aCD3/28) in a concentration-dependent manner. The 2-NBDG (glucose) uptake assay reconfirmed the Mg^2+^@MPP-stimulated high glycolytic capacity (Figures 4c and S25).^47^ Under the effect of Mg^2+^@MPP, the expression of CTL-serial markers, including CD69 (early activation), CD25 (late activation), CD107a (degranulation), and IFN-γ (functional surrogate), were all promoted, substantiating the T-cell modulation capacity of Mg^2+^@MPP (Figures 4d and S26).

The extracellular secretion of TNF-α, IL-2, and IFN-γ, assessed by the enzyme-linked immunosorbent assay (ELISA), was also aggrandized (Figure 4e). Inspired by the eminent enhancement of Mg^2+^@MPP on CTL activation and degranulation, we next examined Mg^2+^@MPP-mediated CTL cytolytic function based on the mismatch between heterologous BALB/c CTLs and C57B/L6J GL261 cancer cells (Figure 4f). The presence of Mg^2+^@MPP significantly influenced the CTL killing performance. 77.99 ± 4.59% of GL261 cancer cells were lysed by Mg^2+^@MPP-stimulated CTLs compared to 62.49 ± 7.64% of the control group, despite at a 2.5:1 ratio (CTLs: GL261), and the former readily increased to 100.55 ± 4.17% at a 10:1 ratio (Figure 4g). Of note, Mg^2+^ ions released from Mg^2+^@MPP animated the phosphorylation of proximal focal adhesion kinase (FAK^Tyr397^) and distal extracellular signal-regulated protein kinase 1/2 (ERK1/2^Thr202/Thr204^) (Figure 4h, i), both of which are downstream signals of T-cell receptor (TCR) and closely associated with the T-cell activity.^48,49^ The phosphorylation of the downstream protein ERK1/2 was reconfirmed by Western blotting analysis, and the results also demonstrated the incapacitation of MK-8931 on T-cell remodeling (Figure S27). Besides, the adiaphorous behavior of MK-8931 on T cells was simultaneously confirmed by FCM analysis (Figure S28). Thus, we demonstrated that an Mg^2+^-based reagent reshaped the CTLs’ cytolytic function *via* a TCR/FAK/ERK_1/2_ signal axis, and these results predicted the potential of Mg^2+^@MK-8931@MPP nanoeditor to mediate metalloimmunotherapy against glioblastoma *in vivo*.

**Figure 5:**
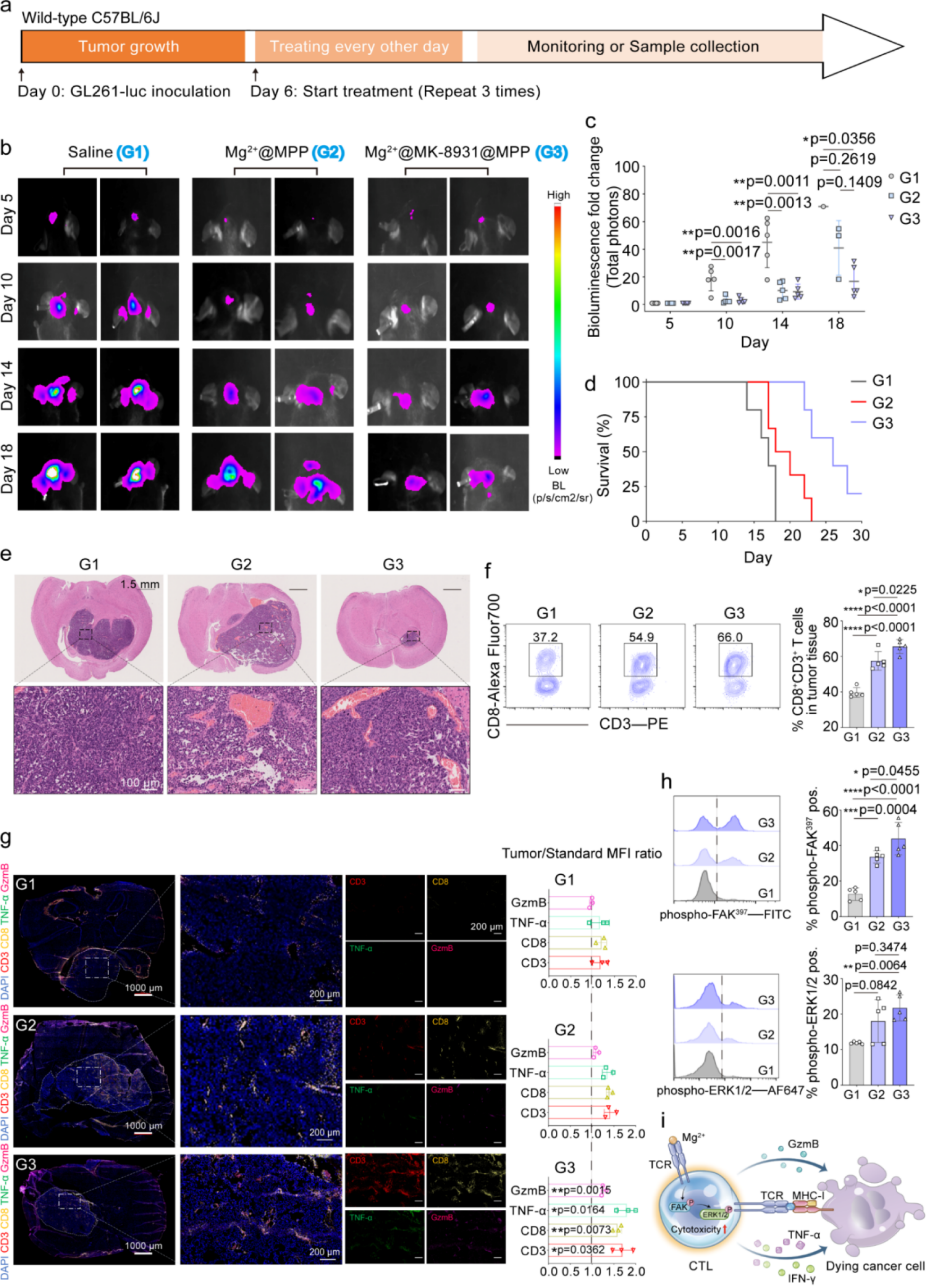
Mg^2+^ cooperating with MK-8931 for CTLs modulation *in vivo*. (a,b,c) Schematic illustration (a), representative bioluminescence images (b), and bioluminescence fold-change analyses (c) of GL261-luc-bearing mice treated with Saline, Mg^2+^@MPP, and Mg^2+^@MK-8931@MPP nanoeditors (MK-8931:6.9 mg per kg of body weight; Magnesium: 22.13 μM per kg of body weight). *n* = 5 per group. (d) Mouse survival per group. (e) H&E staining of mice brains. (f) Representative flow cytometry plots and quantitative analyses of the glioblastoma-infiltrating CTLs phenotypes under diverse treatments. (g) Immunofluorescence (CD3-red, CD8-yellow, TNFα-green, and GzmB-magenta) and zoom-in images of glioblastoma under Saline, Mg^2+^@MPP, and Mg^2+^@MK-8931@MPP treatments. Quantitative analyses on the right displayed the fluorescence intensity ratio of glioblastoma to the background. Value > 1 represents the higher relative abundance of corresponding markers on glioblastoma tissue in comparison with background tissue. The significant differences were calculated by comparing them to the Saline group. (h) Representative histograms and quantitative analyses of the phosphorylation of FAK and ERK1/2 proteins on glioblastoma-infiltrating CTLs. (i) A schematic diagram of Mg^2+^ enhances CTL tumor-killing cytotoxicity by stimulating TCR and its downstream. All flow data presented here were gated on the upper level as shown in Figure S19. Statistical significance was calculated *via* one-way ANOVA with Tukey’s multiple comparisons. *p* values < 0.05 were considered significant: *p < 0.05, **p < 0.01, ***p < 0.001, and ****p < 0.0001.

By treating orthotopic glioblastoma-bearing mice with Mg^2+^@MPP and Mg^2+^@MK-8931@MPP, the function of Mg^2+^ ions in remodeling CTLs can be well studied *in vivo* (Figure 5a). GL261-luc-bearing mice were randomized into 5 numbers per group on Day 6 postinoculation of GL261 cells and received intravenous injections of saline (G1), Mg^2+^@MPP (G2), and Mg^2+^@MK-8931@MPP (G3) every other day, repeated three times. Glioblastoma growth was measured by bioluminescence imaging (Figure 5b,c). Mg^2+^@MPP and Mg^2+^@MK-8931@MPP showed inhibitory action on glioblastoma growth compared to the saline group (G1). The Mg^2+^@MK-8931@MPP-treated group exhibited the relatively weakest bioluminescence, ascribing to the combination of CTL-remodulating Mg^2+^ ions and GAM-polarizing MK-8931. Aligning with the bioluminescent results, Mg^2+^@MK-8931@MPP nanoeditors prolonged mice survival by over 10 days with minimal body weight influence (Figures 5d and S29).

H&E staining identified gliomatous necrosis and apoptosis in the presence of Mg^2+^@MPP (G2) and Mg^2+^@MK-8931@MPP (G3) treatments, and a much-shrunken tumor area highlighting superior efficacy of the latter (Figure 5e). To expound on the foregoing phenomenon, we repeated the therapeutic procedure and harvested tumors and spleen to analyze immunological variations. Unlike the control group (39.72 ± 2.77%), Mg^2+^@MPP enabled 57.46 ± 5.25% of CTLs to infiltrate the glioma tissue. This number could be further increased to 65.58 ± 3.96% by Mg^2+^@MK-8931@MPP (Figure 5f). We next phenotyped glioblastoma-infiltrating CTLs *via* flow cytometric and immunofluorescent analyses. In line with their increase in quantity, both Mg^2+^@MPP and Mg^2+^@MK-8931@MPP promoted the activation (CD69 and CD25) and cytotoxicity (CD107a and IFN-γ) of murine CTLs (Figure S30). These Mg^2+^-treated CTLs secreted more proinflammatory Granzyme B (GzmB) and TNF-α (Figure 5g). To demonstrate their mechanism for functionalizing CTLs *in vivo*, we quantified the phosphorylation of FAK and ERK1/2 as *in vitro* tests. As Figure 5h exhibits, Mg^2+^@MPP and Mg^2+^@MK-8931@MPP highly galvanized the glioblastoma-infiltrating CTLs’ TCR signal and phosphorylated its downstream functional proteins FAK and ERK1/2. All of the above results indicated the capacity of Mg^2+^@MPP and Mg^2+^@MK-8931@MPP in maneuvering T-cell infiltration and cytotoxicity to delay glioblastoma progression (Figure 5i). Since immunological memory is crucial for maintaining long-term immunity,^50^ differentiation of memory T cells (T_em_, CD62L^–^CD44^+^; *T*_cm_, CD62L^+^CD44^+^) was purposefully evaluated. Both T_em_ and *T*_cm_ cells derived from CD4 or CD8 subtypes were appreciably increased under Mg^2+^@MPP and Mg^2+^@MK-8931@MPP treatments (Figure S31).

### aNK1.1 Persists in the CTL Effector Function

**Figure 6:**
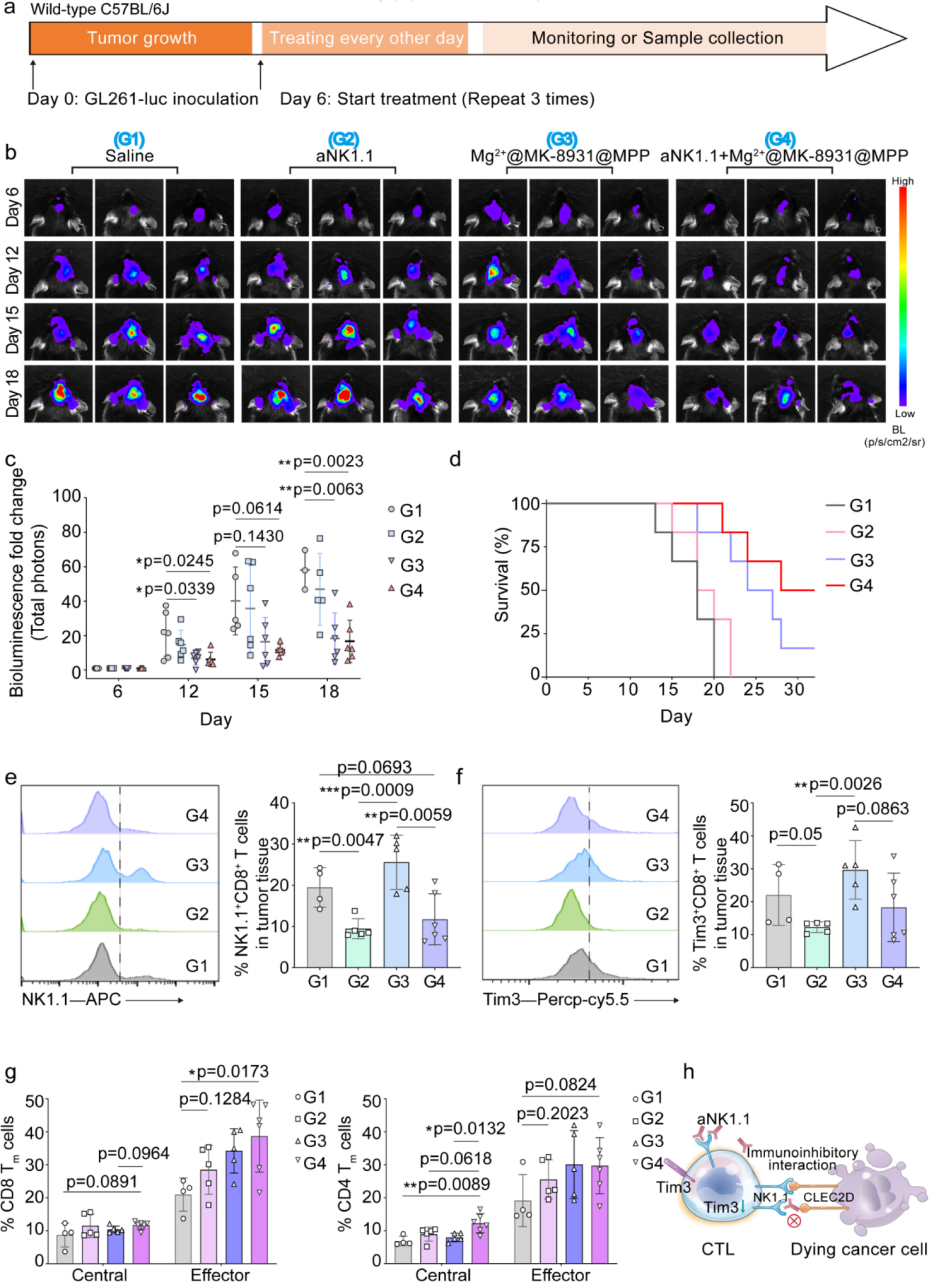
Combination with aNK1.1 to persist CTL killing capacity and decrease CTL exhaustion *in vivo*. (a,b,c) Schematic illustration (a), representative bioluminescence images (b), and bioluminescence fold-change analyses (c) of GL261-luc-bearing mice treated with Saline, aNK1.1, Mg^2+^@MK-8931@MPP, and aNK1.1+Mg^2+^@MK-8931@MPP (MK-8931:6.9 mg per kg of body weight; Magnesium: 22.13 μM per kg of body weight; aNK1.1: 2.5 mg per kg of body weight). *n* = 6 per group. (d) Mouse survival per group. (e,f) Representative histogram and quantitative analyses of NK1.1 and Tim3 expression on glioblastoma-infiltrating CD8 T cells. (g) Quantification of the splenic effector and central memory cells of CD4 and CD8 cells. (h) Graphical illustration of aNK1.1 sustaining CTL killing and decreasing T-cell exhaustion. All flow data presented here were gated on the upper level, as shown in Figure S19. Statistical significance was calculated *via* an unpaired two-tailed *t* test with two-group comparisons. Mouse survival percentage was compared by a log-rank (Mantel-Cox) test. *p* values < 0.05 were considered significant: *p < 0.05, **p < 0.01, ***p < 0.001, and ****p < 0.0001.

Given the recent report that glioblastoma-infiltrating CTLs highly express NK receptors to cause inhibitory interactions with the CLEC2D ligand on glioma cells (Figure S32a), the expression of NK1.1 on corresponding CTLs and CLEC2D on GL261 cells was preferentially determined. The FCM quantified that 23.63 ± 2.58% of GL261 cells expressed CLEC2D ligands

(Figure S32b) and 45.08 ± 4.14% of CTLs possessed NK1.1 marker (Figure S32c). Mg^2+^@MK-8931@MPP therapy, although efficacious, triggered the NK1.1-expressing ratio of CTLs up to 71.8 ± 9.02% at day 7 post-therapy (Figure S32c). Thus, aNK1.1 was proposed to cooperate with Mg^2+^@MK-8931@MPP nanoeditors for combinatorial therapy, sustaining the tumoricidal cytotoxicity of CTLs. The same orthotopic GL261-luc glioblastoma model was established as the aforementioned. We then started the treatment regimen shown in Figure 6a and monitored changes in the bioluminescent signal, body weight, and survival. As envisioned, Mg^2+^@MK-8931@MPP shrank glioblastoma, embodied by the attenuation of the bioluminescent signal and enabled the prolongation of survival, both of which were further enhanced with the aid of aNK1.1 (Figure 6b-d). aNK1.1 exhibited the combinatorial efficacy with Mg^2+^@MK-8931@MPP nanoeditors against glioblastoma. To study the therapeutic function induced by the aNK1.1, we lowered the inoculation dosage of GL261-luc cells (2.5 μL, 2 × 10^5^ cells) to repeat the treatment regimen and collected samples (tumor and spleen) for flow analyses on Day 18. aNK1.1 barely affected the glioblastoma infiltration of CTLs but enhanced the degranulation of CTLs (CD107a positive; Figures S33 and S34). In another dimension, the inhibitory markers of NK1.1 and Tim3 on glioblastoma-infiltrating CTLs were significantly reduced upon aNK1.1-assisted treatment (Figure 6e,f). Figures 6e,f, and S29 reveal the protective performance of aNK1.1 on CTL-sustained killing. Moreover, aNK1.1 combining Mg^2+^@MK-8931@MPP reduced the proportion of T_reg_ cells (Foxp3^+^CD4^+^) by approximately one-fold compared with the Saline group while aggrandized the quantity of antitumor CD4 T cells (Foxp3^–^CD4^+^) (Figure S35). Thus, the killing efficacy of glioblastoma-infiltrating CTLs was consolidated by combinatorial therapy. We subsequently evaluated the long-term immune response potential conferred by combinatorial treatments. As shown in Figure 6g, the addition of aNK1.1 favored the generation of *T*_cm_ and T_em_ cells, no matter from CD4 or CD8 subtypes, which might redifferentiate into effector T cells or show immediate effector function respectively to support antitumor immunity.^51^ All the above *in vivo* results attested to the reshaping capability of Mg^2+^@MK-8931@MPP nanoeditors on CTL cytotoxicity and the maintaining capacity of aNK1.1 for CTL long-term killing (Figure 6h).

### Superiority of Mg^2+^@MK-8931@MPP on T -Cell Modulating Metalloimmunotherapy

**Figure 7:**
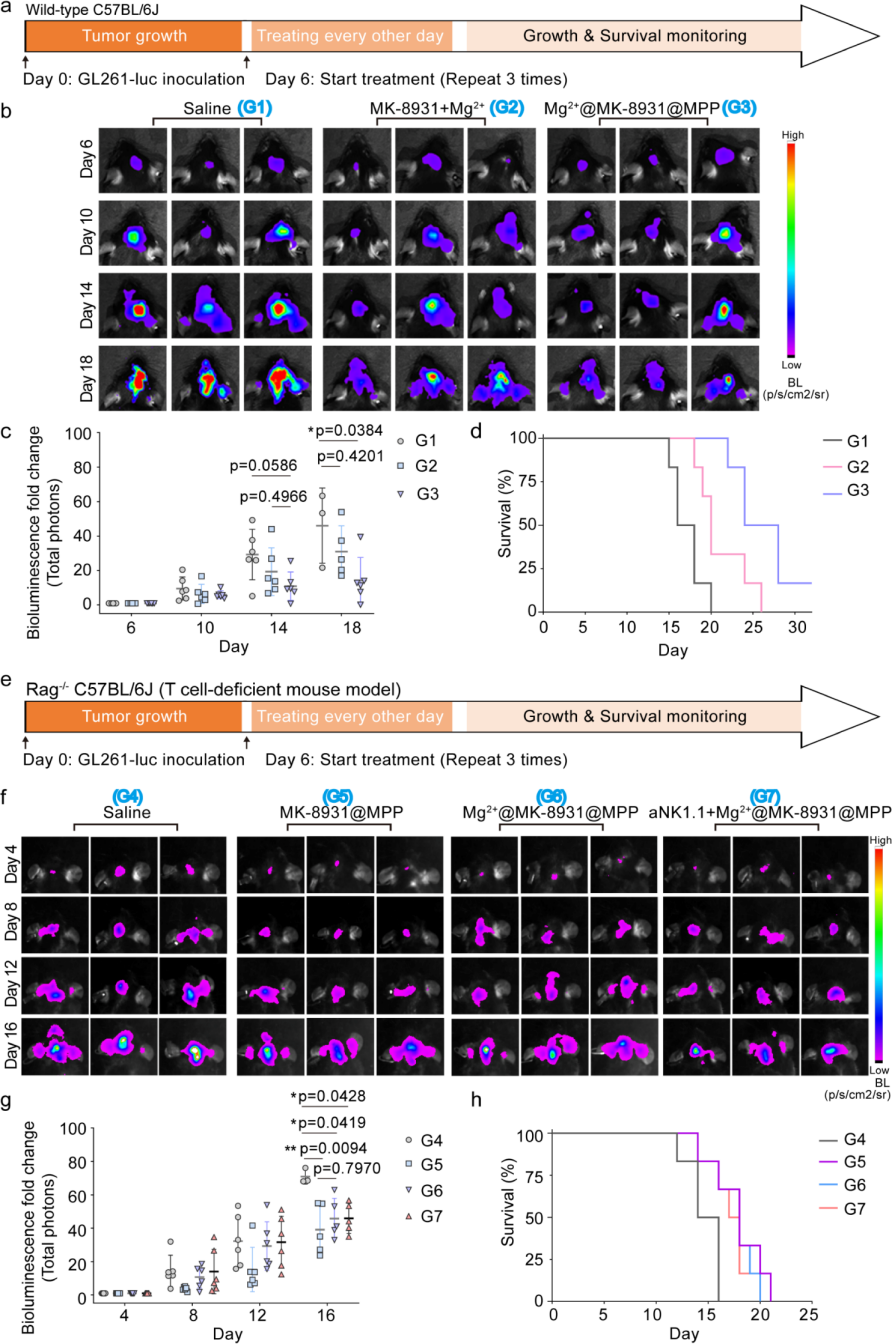
Claiming the superiority of Mg^2+^@MK-8931@MPP nanoeditors in T-cell-modulating metalloimmunotherapy. (a-d) Schematic illustration (a), representative bioluminescence images (b), bioluminescence fold-change analyses (c), and survival rate (d) of GL261-luc-bearing mice treated with Saline, MK-8931+Mg^2+^, and Mg^2+^@MK-8931@MPP (*n* = 6 per group). (e-h) Schematic illustration (e), representative bioluminescence images (f), bioluminescence fold-change analyses (g), and survival rate (h) of GL261-luc-bearing Rag^–/–^ C57B/L6J mice under Saline, MK-8931@MPP, Mg^2+^@MK-8931@MPP, and aNK1.1+Mg^2+^@MK-8931@MPP treatments. Statistical significance was calculated *via* one-way ANOVA with Tukey’s multiple comparisons. Mouse survival percentage was compared by a log-rank (Mantel-Cox) test. *p* values < 0.05 were considered significant: *p < 0.05, **p < 0.01, ***p < 0.001, and ****p < 0.0001.

We then compared the therapeutic effect of Mg^2+^@MK-8931@MPP nanoeditors with free drugs as mentioned in the above sections (Figure 7a). More superior therapeutic efficacy and survival rates were observed in the Mg^2+^@MK-8931@MPP group, claiming that the MPP-fabricated nanoform was beneficial for guaranteeing the T-cell modulating function of Mg^2+^ and MK-8931 (Figure 7b-d).

Next, the T-cell-deficient Rag^–/–^ C57B/L6J mouse was adopted to establish the orthotopic GL261-luc-bearing model for emphasizing the importance of Mg^2+^@MK-8931@MPP and combinatorial aNK1.1 on T-cell modulation. Bioluminescence imaging and survival were monitored every 4 days from Day 4 post-tumor implantation, and the therapeutic loop was initiated on Day 6 (Figure 7e). Finally, modest bioluminescence variations were observed in the MK-8931@MPP, Mg^2+^@MK-8931@MPP, and aNK1.1+Mg^2+^@MK-8931@MPP groups in comparison with those of the saline control group (Figure 7f,g). But different from the significant survival improvement in the immunocompetent mouse model (*p* = 0.0003, aNK1.1+Mg^2+^@MK-8931@MPP versus saline control, Figure 6d), aNK1.1+Mg^2+^@MK-8931@MPP only weakly prolonged the life expectancy of glioblastoma mice in this Rag^–/–^ immunodeficient mouse model (*p* = 0.0382, Figure 7h). These results indicated that MK-8931 repolarizing GAMs inhibited glioblastoma development to a limited degree, and Mg^2+^ and aNK1.1 mainly functioned in CTLs shaping, as evidenced by the indiscriminate glioblastoma inhibition on T-cell-deficient mice across the MK-8931@MPP, Mg^2+^@MK-8931@MPP, and aNK1.1+Mg^2+^@MK-8931@MPP groups. In conclusion, the therapeutic effects on T-cell-deficient Rag^–/–^ and wild-type C57B/L6J mouse models successfully verified the three indispensable elements of aNK1.1+Mg^2+^@MK-8931@MPP in glioblastoma therapy: MK-8931 quantitatively increased CTL infiltration by GAMs polarization to i-GAMs; Mg^2+^ qualitatively enhanced CTL cytolytic function; and aNK1.1 persisted with CTL killing activity.

## Conclusions

In this scenario, we presented Mg^2+^@MK-8931@MPP nanoeditors loaded with MK-8931 and Mg^2+^ to strategically re-educate GAMs and modulate CTL effector function for precise metalloimmunotherapy from the quantitative and the qualitative levels. MPC modification on the MPP polymer permitted the nanoeditor’s superior BBB penetration efficiency, guaranteeing sufficient glioblastoma accumulation. The modificatory polyphenol moieties in the MPP polymer coordinated high-level Mg^2+^ accompanied by the hydrophobic encapsulation of MK-8931 for precise and safe delivery. The glioblastoma-cumulative Mg^2+^@MK-8931@MPP nanoeditors afterward pH-responsively released therapeutic components to launch the tumoricidal cascade reaction: 1) MK-8931 quantitatively promoted i-GAMs-induced CTL infiltration; 2) immune adjuvant Mg^2+^ qualitatively enhanced the CTL effector function; 3) cooperated aNK1.1 sustained CTL killing cytotoxicity. This Mg^2+^@MK-8931@MPP nanoeditor served as a glioblastoma metalloimmunotherapeutic tool for multitiered T-cell modulation, which emphasized that increasing the intratumoral infiltration of CTLs alone does not suffice for glioblastoma treatment; both CTL cytotoxic enhancement and maintenance are equally vital. More importantly, this CTL-modulation nanoeditor has broadened the horizon of glioblastoma treatment to safe-delivered combinatorial metalloimmunotherapy, highlighting a new direction.

## Experimental Methods

### Materials

Supplementary Tables 1–3 list all reagents in this study.

### Fabrication and Characterization of Mg^2+^@MK-8931@MPP Nanoeditor

Mg^2+^@MK-8931@MPP nanoeditor was self-assembled through the chelating effect between Mg^2+^ ions and the phenolic donor MPP polymer under ultrasonication, which entrapped the MK-8931 drug *via* hydrophobic interactions concurrently. In detail, magnesium chloride (MgCl_2_) was dissolved in deionized (DI) water, while MK-8931 and MPP polymer were dissolved in methanol , each at a concentration of 5 mg mL^–1^. Ultrasonically mixed the MgCl_2_ (1.5 mg), MK-8931 (750 μg), and MPP polymer (5 mg) at a mass ratio of 6:3:20 for 20 min, followed by the rotary evaporation of methanol to obtain Mg^2+^@MK-8931@MPP nanoeditor. The Mg^2+^@MK-8931@MPP nanoeditor was purified thrice *via* ultrafiltration centrifugation (4,000 rpm for 30 min). Freeze-dried the products for standby use.

The spheroid morphology and elemental analysis of the Mg^2+^@MK-8931@MPP nanoeditor were detected by High-angle annular dark-field scanning transmission electron microscopy (HAADF-STEM) and energy-dispersive X-ray spectroscopy (EDS, Talos F200S). The lyophilized nanoparticle was collected for X-ray photoelectron spectroscopy (XPS, ESCALAB 250Xi spectrometer) analyses to demonstrate the material composition. The drug loading efficiency and metal encapsulation rate of MK-8931 and Mg^2+^ ions were confirmed by ultraviolet–visible (UV–vis) spectroscopy (SHIMADZU UV-1800) and inductively coupled plasma mass spectrometry (ICP-MS, Thermo Scientific iCAP), respectively. The calculation formula is as follows: Drug loading efficiency (%) = (Encapsulated drug weight)/(Total nanoeditors weight) × 100%; Metal encapsulation rate (%) = (Encapsulated metal quality)/(Original metal quality) × 100%. Nile red instead of MK-8931 was loaded into Mg^2+^@MK-8931@MPP nanoeditors for evaluating the pH responsibility using a fluorescence spectrophotometer (Fluoro Max-4, Horiba, Japan).

### Thermodynamics Analyses by Isothermal Titration Calorimetry (ITC)

The interaction between the polymer (MPP and PP) and MgCl_2_ was measured by isothermal titration calorimeters (MicroCal PEAQ-ITC, Malvern Panalytical). MgCl_2_ solutions were loaded in the injection syringe and then titrated into MPP or PP solutions in the sample cells. Then, the function of the molar ratio of the polymer and MgCl_2_ was plotted by monitoring the molar heat change. Data analysis formula: Δ*G* = Δ*H*-TΔ*S*, *T* = 303 K. Δ*G* < 0 represents the spontaneous reaction and Δ*G* > 0 represents the nonspontaneous reaction.

### *In Vitro* Blood–Brain Barrier (BBB) Penetration

The 0.4-μm pore size transwell membrane (12 mm Transwell, Corning) was equably precoated with Matrigel matrix basement membrane (Corning 254234), and plating mouse brain endothelial cell bEnd.3 cells (5 × 10^4^ cells/well) into the pretreated inserted well for five-day cultivation. The murine stable macrophage cell line RAW264.7 (2 × 10^5^ cells/well) was plated in the lower chamber on the third day. Mg^2+^@MK-8931@MPP, Mg^2+^@MK-8931@PP, and MK-8931@MPP nanoparticles (100 μg of the corresponding polymers) were synthesized with the respective polymers PP-Ce6 and MPP-Ce6, and dispersed in a fresh DMEM medium for further use. When the transepithelial/transendothelial electrical resistance (TEER) reached ca. 150–300 Ω·cm^2^ (EVOM2, World Precision Instruments), the corresponding nanoparticles were added to the upper chamber (with or without 1 h preincubation of HC-3/α-BTX) at 0, 9, and 13 h, finally collecting RAW264.7 cells were collected at 15 h for confocal laser scanning microscopy (CLSM) imaging (Olympus SpinSR10 Spinning Disk Confocal Microscope) and flow cytometry (FCM) analyses (CytoFLEX Flow Cytometer). TEER was calculated using the formula: TEER (Ω × cm^2^) = (Total ohmic resistance (Ω) – Blank resistance (Ω)) × Area of the membrane (cm^2^).

### *In Vivo* Biodistribution Performance of Mg^2+^@MK-8931@MPP Nanoeditor

To evaluate the biodistribution and metabolism state of the Mg^2+^@MK-8931@MPP nanoeditor, we established the orthotopic GL261-luc glioblastoma C57B/L6J model and randomized it into 4 groups (3 mice per group) at day 9. Then, the Ce6-visualized Mg^2+^@MK-8931@MPP, Mg^2+^@MK-8931@PP, and MK-8931@MPP nanoparticles (containing 50 μg of Ce6) were intravenously injected for fluorescence imaging at diverse time points (3, 6, 12, and 24 h). For group 4, the nAChRs inhibitor (α-BTX) and ChTs inhibitor (HC-3) were preinjected to block MPC function sites. Meanwhile, we repeated model establishment and injected Mg^2+^@MK-8931@MPP nanoeditors to collect major organs (heart, liver, spleen, lung, kidney) and brain tumors at diverse time points (3, 6, 12, 24 h) after saline wash to detect their magnesium concentrations by ICP-MS after the digestion of fresh aqua regia (concentrated nitric acid:concentrated hydrochloric acid = 1:3, v/v). The actual magnesium concentration was calculated by deducting the original magnesium in animal tissues.

### *In Vitro* Assays of Murine CTLs

#### Measurement of Glycolytic Activity

A seahorse XF96 extracellular flux analyzer (Agilent Technologies) was exploited to determine the function of Mg^2+^ on the metabolic profile (glycolytic activity) of murine CTLs. Murine naïve CD8 T cells were isolated from splenocytes of C57BL/6J mice and subsequently activated by anti-CD3/-CD28 antibodies (aCD3/28) to obtain murine CTLs. CTLs were plated (10^5^ cells/well) onto a poly-d-lysine-precoated XF96 cell plate. A Mg^2+^ solution (0, 0.4, 0.8, 1.2, 1.6 mM) with XF base medium containing 2 g L^–1^ glucose was added into the CTLs plates. Notably, aCD3/28 were applied onto plated cells *via* the instrument’s multi-injection port from the beginning of this assay to reachfinal concentrations of anti-CD3 antibodies (5 μg mL^–1^) and anti-CD28 antibodies (2.5 μg mL^–1^) per well for maintaining murine CTL activation. Real-time monitoring of ECAR (mpH/min) was recorded to subtract baseline ECAR for further analysis.

#### Glucose Uptake Assay

The fluorescent glucose analog 2-NBDG was utilized to evaluate the glucose uptake status of CTLs treated by Mg^2+^ ions or Mg^2+^@MPP nanoparticles. Murine CTLs were activated with aCD3/28 or left untreated for 12 h and seeded (2 × 10^5^ per well) into a 96-well plate, followed by the addition of Mg^2+^ ions or Mg^2+^@MPP nanoparticles at final Mg^2+^ concentrations of 0.4, 0.8, and 1.2 mM for 12 h of coculture. The original medium was replaced with a glucose-free medium containing 2-NBDG (10 μM) for 30 min, and then the treated CTLs were collected for flow cytometry analysis.

#### Murine CTL Activation and Cytotoxicity Detection

Murine CTLs were activated with anti-CD3 antibodies (3 μg mL^–1^) and anti-CD28 antibodies (1 μg mL^–1^) before treatment of Mg^2+^ (1.2 mM) and Mg^2+^@MPP nanoparticles. For the detection of surface activation markers (early activation: CD69, late activation: CD25), CTLs were stimulated by aCD3/28 for 3 h and treated for 6 and 14 h, respectively. For the detection of intracellular markers (degranulation marker: CD107a, cytotoxic marker: IFN-γ), CTLs were sustained for 8 h stimulation of aCD3/28 and maintained to the end of treatment about 16 h, and finally were fixed and permeabilized prior to the cell staining. To analyze ERK1/2 phosphorylation and FAK phosphorylation, CTLs were stimulated for 3 h and treated for 6 h, and fixation and permeabilization were also necessary here.

#### Murine CTL Cytotoxicity Assay

CTL cytotoxic remodeling was studied by measuring lactate dehydrogenase (LDH, an indicator of cytotoxicity) with the CytoTox 96 Nonradioactive Cytotoxicity Assay Kit (Promega). Naïve CD8 T cells, isolated from BALB/c mice, were activated by aCD3/28 for 48 h and, afterward, proliferated for 3–4 days under stimulation of IL-2 (100 IU mL^–1^). Prior to the coincubation of CTLs and GL261, CTLs treated with1.2 mM concentration of Mg^2+^ and Mg^2+^@MPP for 4 h, subsequently a CTL-GL261 coculture system was estabilished at different ratios in the presence of 10 ng mL^–1^ PMA and 0.5 μM ionomycin for 4 h. Finally, the percentage of GL-261 cell lysis was quantified by a nonradioactive colorimetric assay.

### Mouse Orthotopic GL261-luc Glioblastoma Models

C57BL/6J or Rag1^–/–^ C57BL/6J mice were first anesthetized with 1.25% tribromoethanol dissolved in tertiary amyl alcohol and immobilized by a stereotaxic instrument (RWD Life Science), followed by the intracranial injection of GL261-luc cells (5 μL, 4 × 10^5^) into the right striatum parenchyma at the designated location (anterior to bregma: 1.5 mm, right lateral: 2.0 mm, depth: 3 mm). The inoculation dosage of GL261-luc cells was reduced by half when conducting the immunological evaluation experiments and monitoring the growth status of intracranial glioma cells by bioluminescent imaging (IVIS imaging system, Bruker Xtreme 4MP system, or BLT AniView100) to determine a suitable therapeutic time. Mice were randomized at day 6 after tumor inoculation, and they continued to receive various treatments intravenously according to detailed experiments. Repeat the treatment every other day three times and maintain monitoring at three additional time points.

### Statistical Analysis

GraphPad Prism version 8.0.2 was used for data analysis. Unless otherwise indicated, all quantitative data are shown as the mean ± standard deviation (SD). For statistical differences determination, an unpaired/paired two-tailed *t*-test was used for two-group comparisons and one-way ANOVA with Tukey’s multiple comparisons for more than two groups. Mouse survival percentage was compared by a log-rank (Mantel-Cox) test. Replicate numbers (*n*) for each experiment are indicated in Figure legends. *p* values < 0.05 were considered significant: *p < 0.05, **p < 0.01, ***p < 0.001, and ****p < 0.0001.

## Supporting Information Available

Materials, synthesis methods, synthetic route, additional material characterization, and *in vitro*/*in vivo* experimental results, including ^1^H NMR, nanoparticle stability, isothermal titration calorimetry, UV–vis spectroscopy, and representative flow cytometry plots and quantitative analyses (PDF)
