# Supplementary material for: Magnesium–Phenolic Nanoeditor Refining Gliomatous T Cells for Metalloimmunotherapy
Source: ACS Nano. 2024 Dec 19;19(1):1222–37. doi: 10.1021/acsnano.4c13388 (PMC11752515; doi:10.1021/acsnano.4c13388)
Supplement: Supplementary file 1 — nn4c13388_si_001.pdf [file nn4c13388_si_001.pdf]

Magnesium-phenolic nanoeditor refining gliomatous T cells for metalloimmunotherapy

Wenxi Li,[1,2,#] Hao Tian,[1,2,#] Ziliang Yan,[1,2] Xinying Yu,[1,2] Bei Li,[1,2,*] and Yunlu Dai[1,2,*]

[1]Cancer Centre and Institute of Translational Medicine, Faculty of Health Sciences, University of Macau, Macau SAR 999078, China.

[2]MoE Frontiers Science Center for Precision Oncology, University of Macau, Macau SAR 999078, China.

*To whom correspondence should be addressed. Tel: (+) 853-8822 4881, Fax: (+) 853-8822 2314; E-mail: yldai@um.edu.mo (Y.D.); beili@um.edu.mo (B.L.)

[#]These authors contributed equally to this work.

Experimental sections

Synthesis of MPC-PEG-polyphenol (MPP) polymer

Synthesis of MPC-PEG. Firstly, the RAFT agent CBPA (14.0 mg, 0.05 mmol), Poly(ethylene glycol) methyl ether methacrylate (average molecular weight ~475, 475.0 mg, 1.0 mmol), MPC (59.0 mg, 0.2 mmol) and AIBN (13 mg, 78 μmol) were dissolved in 1.5 mL CH_3OH and bubbled with nitrogen, then the mixture was stirred in an oil bath at 60 ℃ for 12 hours to perform reversible addition-fragmentation chain transfer (RAFT) polymerization. After the reaction, the products were precipitated in diethyl ether 3 times and the residues were dried in a vacuum oven overnight (494 mg).

Synthesis of MPC-PEG-NHBoc. The intermediate MPC-PEG (315 mg) was further polymerized with amino-functionalized monomer (tert-butyl (2-methacrylamidoethyl) carbamate, 228 mg, 1.0 mmol) that synthesized the same as our previous work. Both agents and AIBN (14 mg, 84 μmol) were dissolved in 1.5 mL CH_3OH and bubbled with nitrogen. Subsequently, RAFT polymerization was carried out in an oil bath at 60 ℃. After 12 h, the

mixture solution was concentrated and precipitated into an excess of diethyl ether, then the residues were dried overnight at room temperature (528 mg).

Synthesis of MPC-PEG-NH_2. MPC-PEG-NHBoc (400 mg) was dissolved in the solution (3 mL CH_2Cl_2, 1 mL CH_3OH, 1 mL TFA) and stirred for 6 h. All solvents were evaporated using a vacuum rotary. The products were redissolved in 30 mL CH3OH, concentrated to 1 mL, and precipitated into excess diethyl ether to obtain pure MPC-PEG-NH_2 (298 mg).

Synthesis of MPC-PEG-polyphenol (MPP). MPC-PEG-NH_2 (250 mg) and 3,4-dihydroxybenzaldehyde (202 mg, 1.5 mmol) were dissolved in 20 mL MeOH and reacted under nitrogen at 60 ℃ overnight to obtain the final product MPP (257 mg).

Synthesis of the PP/MPP-IR780. The polymer for imaging experiments with fluorescent dyes was synthesized by the method reported in our previous work. MPP (200 mg) and IR 780 (15 mg, 22.5 μmol) were dissolved in DMSO. Triethylamine (50 μL) was added to the solution and stirred for 3 days at room temperature. Subsequently, the reaction was dialyzed for two days (MWCO, 3.5 kDa) in the dark against distilled water, and then the solution was lyophilized to produce a solid product (128 mg). MPC-PEG-polyphenol-IR780 (MPP-IR780) and PEG-polyphenol-IR780 (PP-IR780) were synthesized for experiments involving fluorescent imaging.

Magnesium measurements with ICP-MS

The magnesium coordinating rate, pH-responsive release profile in vitro, and biodistribution performance of magnesium coordinated Mg^{2+}@MK-8931@MPP nanoeditor were detected with ICP-MS upon specific processing. For measuring the pH-responsive release of magnesium from Mg^{2+}@MK-8931@MPP, the Mg^{2+}@MK-8931@MPP nanoeditors were incubated with solutions at pH 7.4 or pH 6.5 in Slide-A-Lyzer™ MINI Dialysis Device (3.5K MWCO, 2 mL). The lower layer solution was regularly collected and digested with aqua regia and then diluted with 2% nitric acid to a suitable concentration for detection.

Cells and animals

Preparation of M2-like BMDMs. The hind limbs of 70% alcohol-sterilized C57BL/6J mice (8-10 weeks old) were cut to obtain the femurs and tibias by removing surrounding muscular tissue. After the sterilization of bones, clipped the bone ends and flushed the bones repeatedly with

serum-free 1640 medium to harvest bone marrow cells. Bone marrow monocytes were then purified by depleting red blood cells using an ACK lysing buffer. Whereafter resuspended and cultured the cells by Roswell Park Memorial Institute (RPMI) 1640 medium containing 10% 56℃ hyperthermia inactivated fetal bovine serum (FBS), 1% penicillin-streptomycin, 1 mM sodium pyruvate, 0.1 M HEPES, and macrophage colony-stimulating factor (M-CSF,100 ng mL^{-1}) about 7d to differentiate bone marrow monocytes into BMDMs, further differentiating into M2-like BMDMs under the presence of Interleukin-4 (IL-4, 20 ng mL^{-1}) for 24h.

Isolation of murine naïve CD8 T cells. Spleens from 70% alcohol-sterilized C57BL/6J mice (8-10 weeks old) were extracted and triturated into a single-cell suspension, followed by isolation of naïve $CD8^+$ T cells by a magnetic bead-based isolation kit (EasySep™ Mouse $CD8^+$ T cell isolation kit, Stemcell). The naïve $CD8^+$ T cells were then cultured by RPMI 1640 medium containing 10% 56℃ hyperthermia inactivated FBS, 1% penicillin-streptomycin, 1 mM sodium pyruvate, 0.1 M HEPES, and 50 μM β-mercaptoethanol. The activation of naïve $CD8^+$ T cells for transforming to cytotoxic T cells (CTLs) was stimulated by anti-CD3 antibodies (5 μg mL^{-1}) and anti-CD28 antibodies (2.5 μg mL^{-1}), which appended interleukin-2 (IL-2, 100 IU mL^{-1}) for proliferation promotion.

Cell culture. Semi-adherent macrophage RAW264.7 was cultured in RPMI 1640 medium containing 10% FBS and 1% penicillin-streptomycin. Murine BMDMs and CD8 T cells were cultured by specifically configured RPMI 1640 medium mentioned in the above methods. Luciferase-expressing murine glioma 261 (GL261-luc) cells and mouse brain endothelial bEnd.3 cell lines were cultured by Dulbecco’s Modified Eagle’s Medium (DMEM) containing 10% FBS and 1% penicillin-streptomycin. All cell lines we use are confirmed to be mycoplasma-free by detection kits (Thermo Fisher, M7006) and cultured in a 37 °C incubator with 5% carbon dioxide.

Animals. C57BL/6J mice (4-6 weeks) and $Rag1^{-/-}$ C57BL/6J mice (4-6 weeks) were obtained from the Animal Research Core of the Faculty of Health Sciences, University of Macau (UM). The animal research experiments were subjected to the ethical guidelines (protocol ID: UMARE-030-2021 and UMARE-031-2022) approved by the Animal Ethics Committee at UM. All mice were bred in the specific pathogen-free (SPF) animal facility of UM.

Differentiation and Repolarization of Bone marrow-derived macrophages (BMDMs)

Free MK-8931 drug (0, 25, 50 μg mL^{-1}) was used to explore its inhibitory effect on M2 polarization, and IL-4 (40 ng mL^{-1}) was selected as a positive control. Treated BMDMs were collected after 24 hours of treatment for CD206 and F4/80 staining, followed by FCM analysis. Free MK-8931 drug and MK-8931@MPP (MPP polymer self-assembled nanoparticles loading MK-8931) with a concentration of 50 μg mL^{-1} were employed to confirm its effect on the promotion of M1-like BMDMs and their phagocytic ability to GL261 cancer cells, pre-treated BMDMs and GL261 were predyed by CMFDA and CMTPX, respectively. Lipopolysaccharide (LPS, 50 ng mL^{-1}) was set as the positive control. After 24 or 8 h, the treated BMDMs were collected for FCM analysis.

In vivo efficacy against orthotopic GL261-luc glioblastoma

Mouse orthotopic GL261-luc-bearing C57B/L6J or $Rag^{-/-}$ C57B/L6J model was established for therapeutic effect study. At day 6 post-tumor implantation, we randomized the mice into 5 or 6 per group and initiated the treatment loop in accordance with the experimental demand. Analysis of the bioluminescent intensity and survival time to estimate the efficacy of Mg^{2+}@MK-8931@MPP nanoeditors and the corresponding combinatorial therapeutic effect with anti-NK1.1 antibody (aNK1.1).

MK-8931@MPP repolarizes glioblastoma-associated macrophage (GAMs) and promotes T cell infiltration. In this animal experiment, we set up Saline, free MK-8931, and MK-8931@MPP (6.9 mg MK-8931 per kg body weight) groups to verify whether MK-8931 can promote the repolarization of macrophages into an M1-like type, thereby promoting the infiltration of T cells into the glioblastoma microenvironment. Started therapy on the 6th day in intravenous injection and repeated the treatment thrice every other day. We kept the tumor growth monitoring by bioluminescent IVIS imaging on the 5th, 10th, 14th, and 18th days, as well as the mice survival times.

Mg^{2+}@MK-8931@MPP remodels T-cell cytotoxicity. PBS, Mg^{2+}@MK-8931@MPP (MPP polymer self-assembling with Mg^{2+}), and Mg^{2+}@MK-8931@MPP groups were set in this animal experiment to investigate their capability of reshaping T-cell cytotoxicity and glioblastoma elimination. After randomizing the GL261-luc-bearing mice, mice were treated

intravenously with calculated doses of magnesium (22.13 μM per kg body weight) and MK-8931 (6.9 mg per kg body weight) on day 6, which was repeated three times every other day. Tumor growth monitoring was maintained by bioluminescent IVIS imaging on the 5th, 10th, 14th, and 18th days, while mouse survival time was recorded.

Combinatorial therapy of Mg^{2+}@MK-8931@MPP and anti-NK1.1 antibody. To investigate whether an aNK1.1 could improve T cell exhaustion and enhance glioma growth inhibition, the efficacy of an aNK1.1 was thence evaluated by synergistically combining with Mg^{2+}@MK-8931@MPP. The randomized mice (n = 6) were first treated with Mg^{2+}@MK-8931@MPP as in the above animal experiment, followed by the intravenous injection of aNK1.1 (2.5 mg per kg body weight) 2 days later. Repeat the treatment every other day for three repetitions. Tumor growth monitoring was maintained by bioluminescent IVIS imaging on the 6th, 12th, 16th, and 20th days, while mouse survival time was recorded.

Verifying the importance of macrophage polarization and T cell cytotoxicity. Benefited from the T-cell lacking property of immunodeficient $Rag^{-/-}$ C57B/L6J mice, mouse orthotopic GL261-luc-bearing $Rag^{-/-}$ C57B/L6J model alternative to C57B/L6J was used to confirm the importance of specific remodeling of macrophage and T cells. The same randomized and treated procedures were conducted but changed the groups to PBS, MK-8931@MPP, Mg^{2+}@MK-8931@MPP, and Mg^{2+}@MK-8931@MPP+aNK1.1. Tumor growth was continuously monitored by bioluminescence imaging (IVIS Imaging System, BLT AniView100), and survival was also recorded.

Collection of Treated Samples. All the above animal experiments (n = 6, 7) were repeated, and corresponding samples (glioblastoma tissue, lymph nodes, and sera) were collected at specific time points (MK-8931@MPP and Mg^{2+}@MK-8931@MPP models for 13 days, aNK1.1+Mg^{2+}@MK-8931@MPP model for 18 days) for further analysis, including FCM, CLSM, and biochemistry analyses. The collected tumors and lymph nodes for cytometry analysis should first be minced into small pieces thoroughly by sterile scalpels, then adequately digested by the DMEM medium containing 1 mg ml^{-1} of collagenase type IV and 10 μg ml^{-1} DNase I at a 37℃ incubator for about 45 mins, and finally filtered through a 70-μm strainer to obtain a single-cell suspension. The single-cell suspension of corresponding samples was fixed

and permeabilized by Flow Cytometry Permeabilization/Wash Buffer I after the live/dead staining (LIVE/DEAD™ fixable near-IR dead cell stain kit, Thermo Fisher Scientific), ultimately redispersed into the FACS buffer (PBS + 2% FBS). Sera for biochemistry analyses were kept at room temperature for at least 60 mins to allow clot formation and subsequently centrifuged at 12, 000 rpm for 15 mins at 4 °C, only collecting the supernatant for further use.

Flow cytometric analysis of immune cells

Processed single-cell suspensions were pre-blocked with 0.5 μg anti-mouse CD16/32 monoclonal antibodies (Fc blocker) per million cells for 10 min before immunostaining to exclude Fc-mediated non-specific staining reactions. To analyze the population of tumor-infiltrating immune cells, cells were sequentially stained with fluorescein-labeled anti-mouse antibodies including CD45, CD3e, CD4, CD8a, NK1.1, CD69, CD107a, CD25, IFNγ, phos-FAK, phos-ERK1/2, CD44, and CD62L for T cells analyses, also CD11b, IBA1, FIZZ1, and iNOS for tumor-associated macrophages analyses according to the manufacturer's instructions. For lymphoid immune cells, stained with fluorescein-conjugated anti-mouse antibodies, including CD11c, CD80, and CD86 for dendritic cells, and CD3, CD8, and Ki67 for CD8 T cells. All cytometric analyses were conducted on a CytoFLEX S flow cytometer (Beckman) and analyzed by FlowJo software version 10.0.7.

In Vivo Bioluminescent Imaging

Based on the luciferase labeling of GL-261, 100 μl bioluminescent substrate VivoGlo™ D-Luciferin (P1043, Promega) with a concentration of 8 mg ml^{-1} was intraperitoneally injected into Glioblastoma-bearing mice for bioluminescent imaging of tumor growth. Whereafter the mice were anesthetized with 1.25% tribromoethanol dissolved in tertiary amyl alcohol 8 mins post D-Luciferin injection and transferred to the dark chamber of IVIS imaging system (Bruker Xtreme 4MP system or BLT AniView100) for bioluminescent imaging. The imaging pattern is the "Luminescence" modality and "Reflectance" background image. Data were subsequently analyzed using Bruker MI imaging software version 7.5.2.22464 or BLT AniView100 software version 1.00.00680.

Blood sera detection

Secreted levels of cytokines in serum including Interleukin-10 (IL-10), IL-12, Interferon-γ (IFN-γ), tumor necrosis factor-alpha (TNF-α), and IL-2 were quantified by enzyme-linked immunosorbent assays (ELISA) according to the manufacturer's Instructions. The biosafety of Mg^{2+}@MK-8931@MPP nanoeditor was evaluated by detecting typical biochemical markers, including blood urea nitrogen (BUN), creatinine (CRE), aspartate transaminase (AST), and alanine aminotransferase (ALT).

Statistical analysis

GraphPad Prism version 8.0.2 was used for data analysis. Unless otherwise indicated, all quantitative data are shown as the mean ± standard deviation (SD). For statistical differences determination, an unpaired/paired two-tailed t-test was used for two-group comparisons and one-way ANOVA with Tukey's multiple comparisons for more than two groups. Mouse survival percentage was compared by a log-rank (Mantel-Cox) test. Replicate numbers (n) for each experiment are indicated in Figure legends. P values < 0.05 were considered significant: *P < 0.05, **P < 0.01, ***P < 0.001, and ****P < 0.0001.

Supplementary Figures

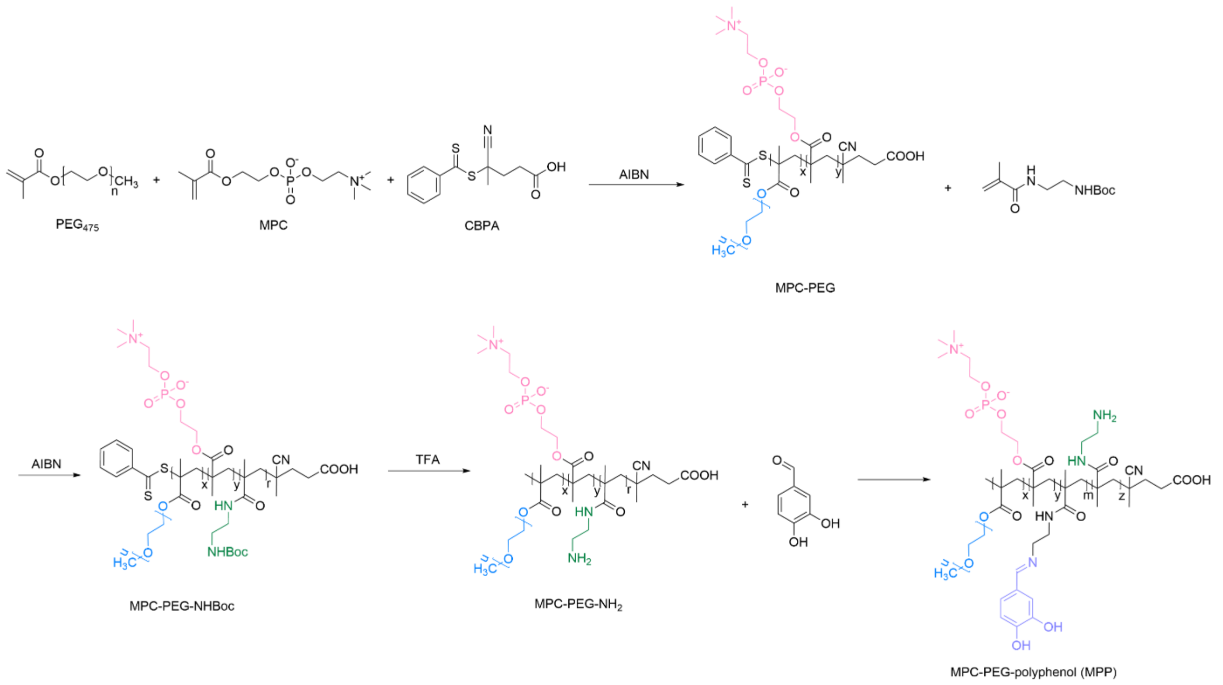

Figure S1. The synthesis route of pH-responsive MPC-PEG-polyphenol (MPP) polymer.

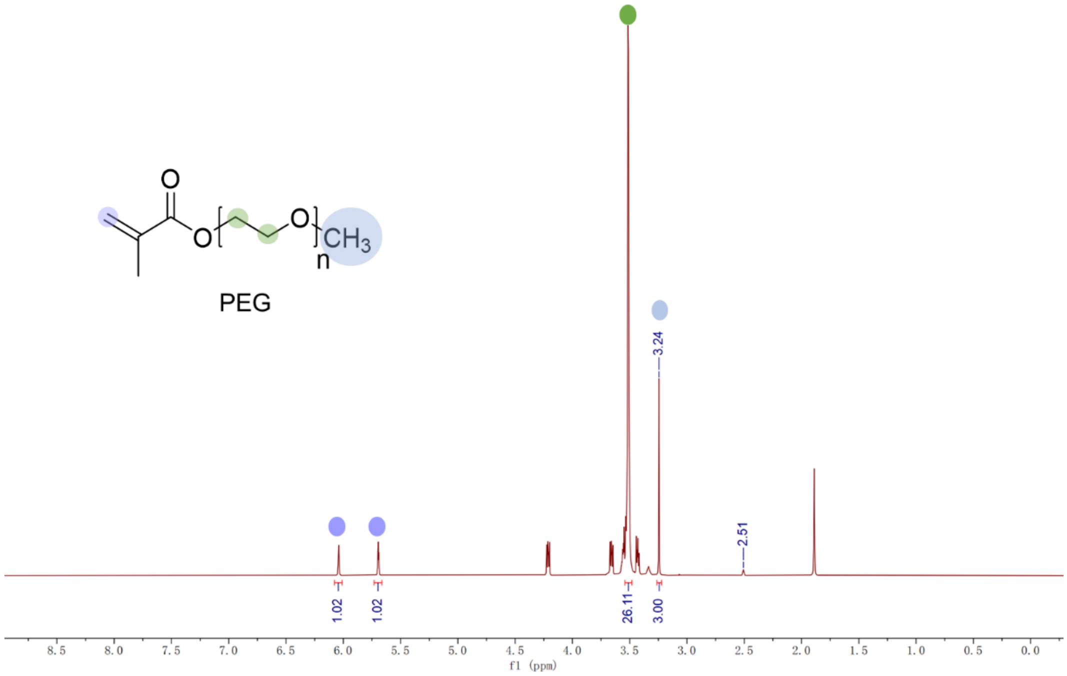

Figure S2. ^{1}H NMR spectrum of the PEG_{475} in DMSO-d6.

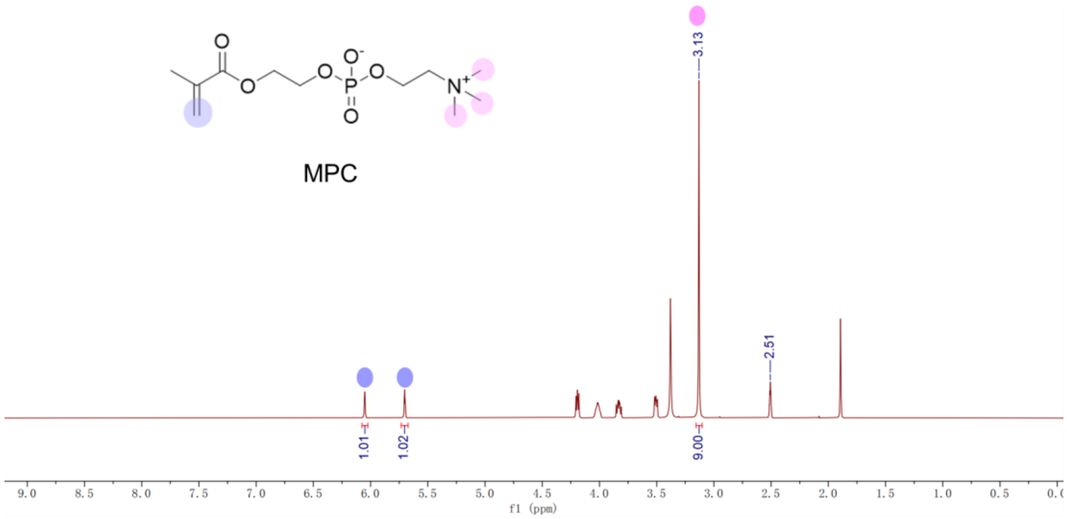

Figure S3. ^{1}H NMR spectrum of the MPC in DMSO-d6.

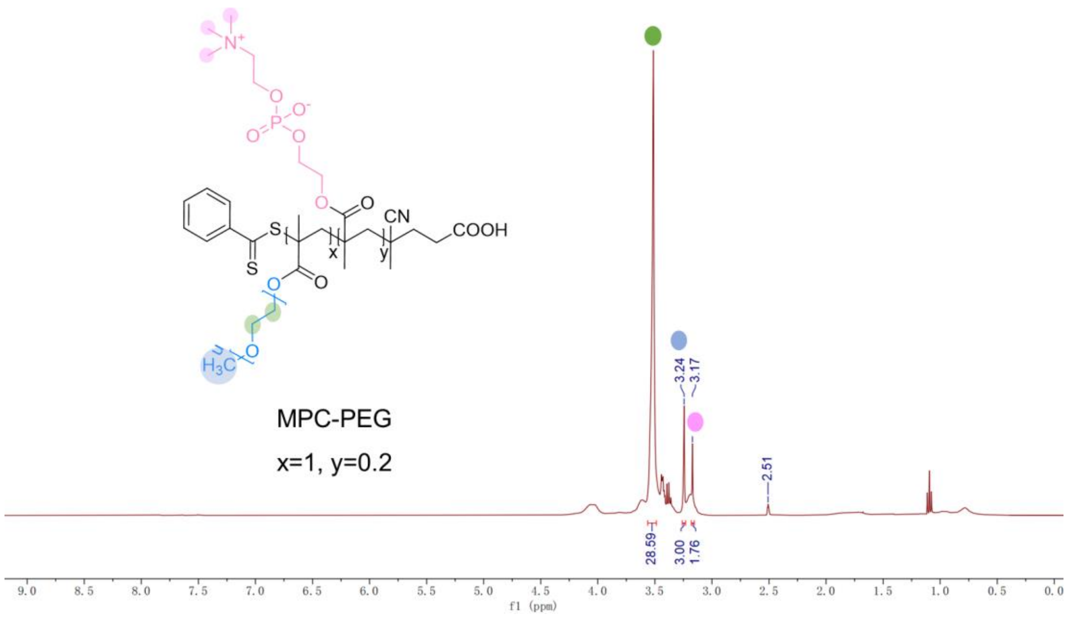

Figure S4. ^{1}H NMR spectrum of the MPC-PEG in DMSO-d6.

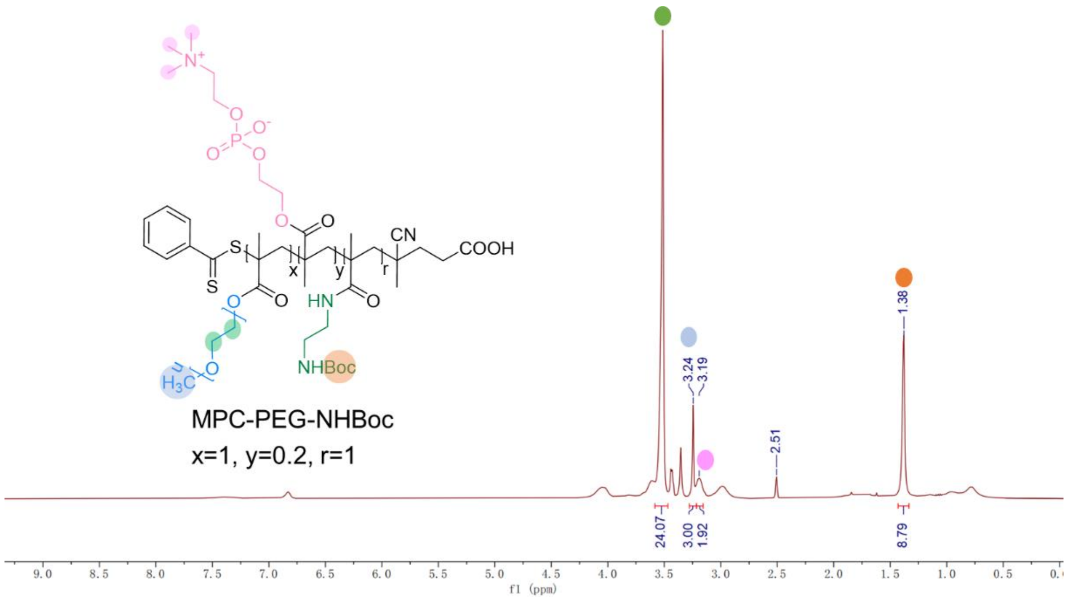

Figure S5. [1] H NMR spectrum of the MPC-PEG-NHBoc in DMSO-d6.

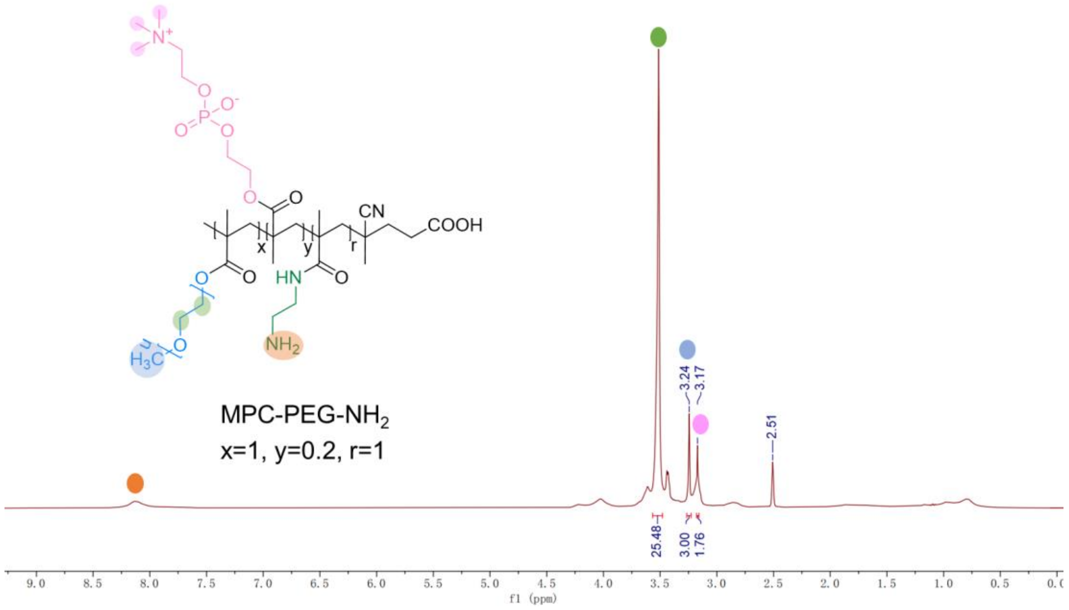

Figure S6. ^{1}H NMR spectrum of the MPC-PEG-NH_2 in DMSO-d6.

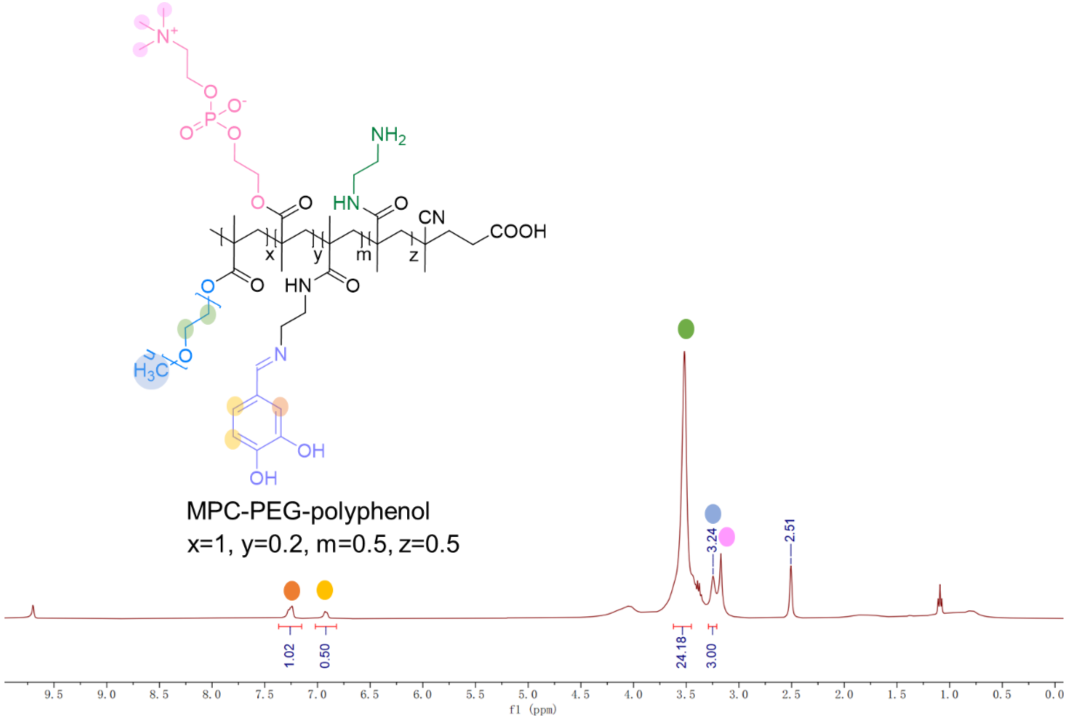

Figure S7. ^{1}H NMR spectrum of the MPC-PEG-polyphenol (MPP) polymer in DMSO-d6.

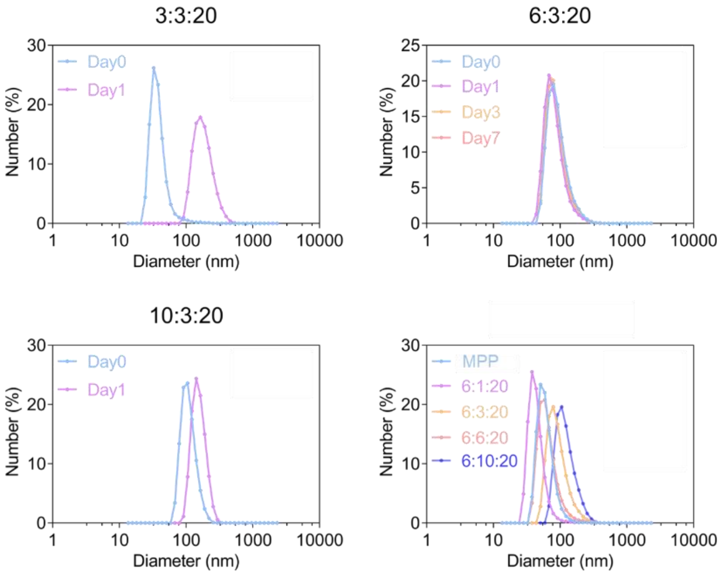

Figure S8. The dynamic light scattering (DLS) size of nanoeditors with diverse weight ratios (Mg^{2+}: MK-8931: MPP) in ddH_2O on days 0, 1, 3, and 7 stored at 4℃.

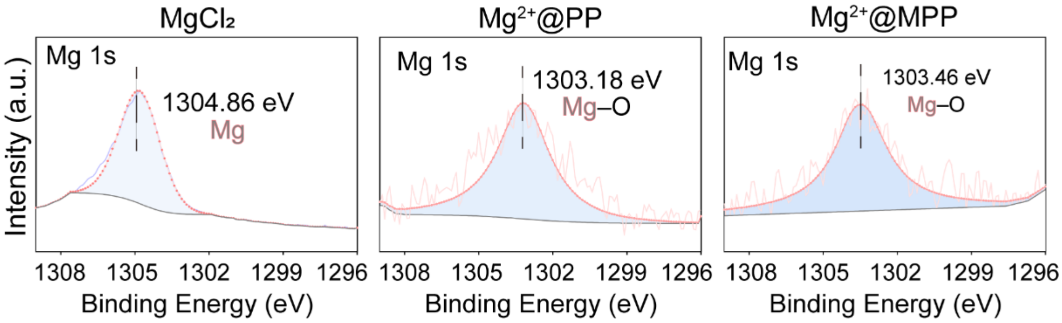

Figure S9. Fit curves and assign photoelectron peaks (Mg 1s) in X-ray photoelectron spectroscopy (XPS) spectra of PP, Mg^{2+}@PP, MPP, and Mg^{2+}@MPP.

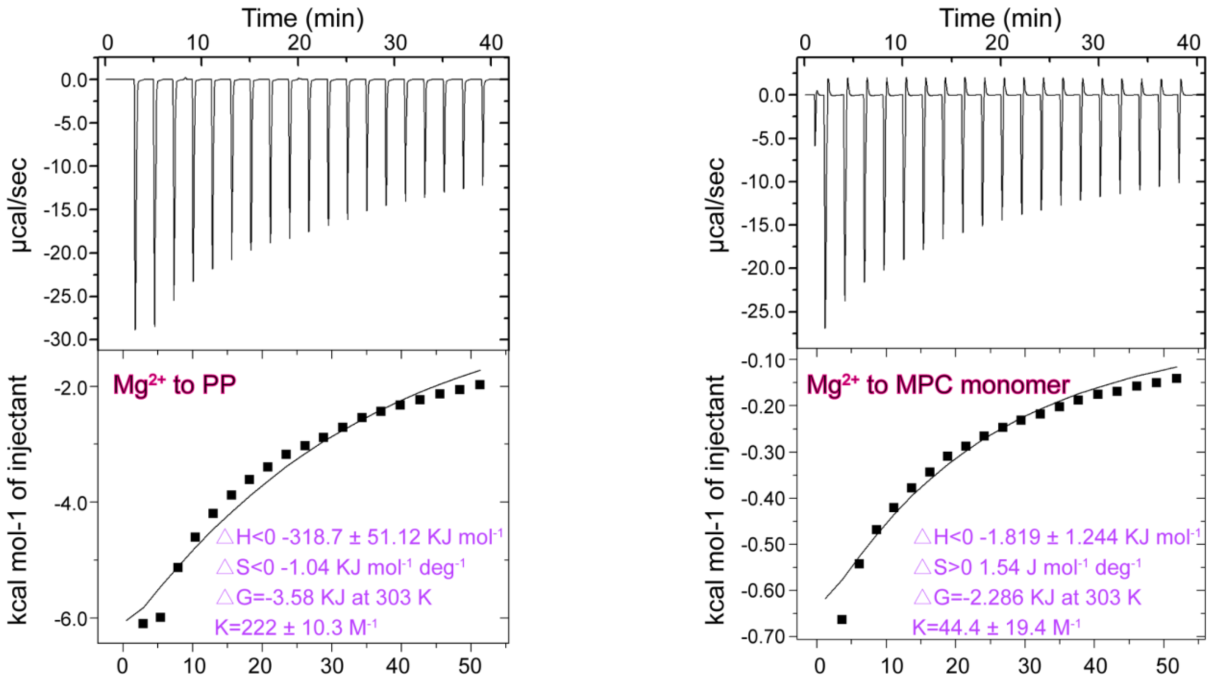

Figure S10. Thermodynamic ITC curve obtained by titration of $MgCl_2$ in PP solution or MPC monomer at 29.85°C (means T=303 K). The top panels represent the raw data, and the bottom panels represent the fitted curves of molar heat changes upon the different PP-to-$MgCl_2$ or MPC-to-$MgCl_2$ molar ratios. The ΔG (ΔG=ΔH-TΔS, T is kelvin temperature) of PP-to-$MgCl_2$ (-3.58 KJ) was similar to MPP-to-$MgCl_2$ (-3.236 KJ, shown in Figure 1g), and both lower than MPC-to-$MgCl_2$ (-2.286 KJ), revealing the stronger spontaneous interaction between $MgCl_2$ and PP/MPP. The small K value in the MPC-to-MgCl2 group reflected negligible interaction between MgCl2 and MPC, much less the MPC moiety in MPP polymer subjected to the steric-hindrance effect.

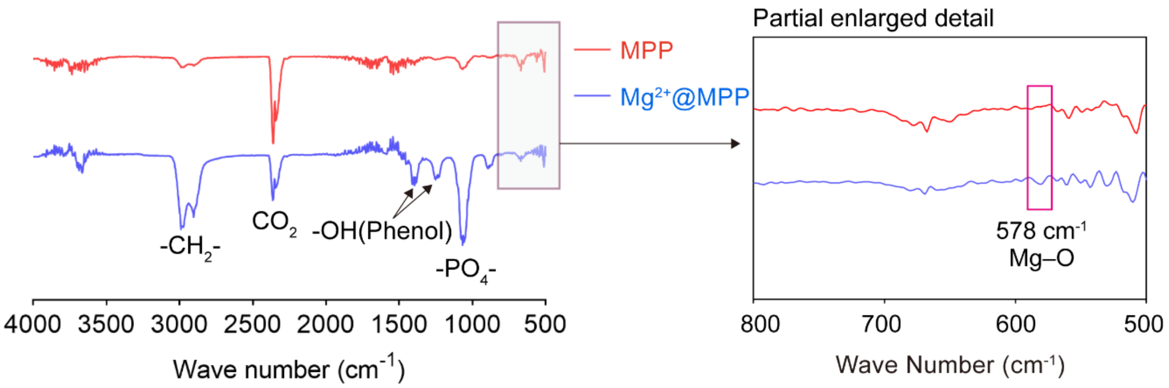

Figure S11. FTIR spectra of MPP and Mg^{2+}@MPP. The peak at 578 cm^{-1} in the Mg^{2+}@MPP group indicates the Mg–O bond formation.

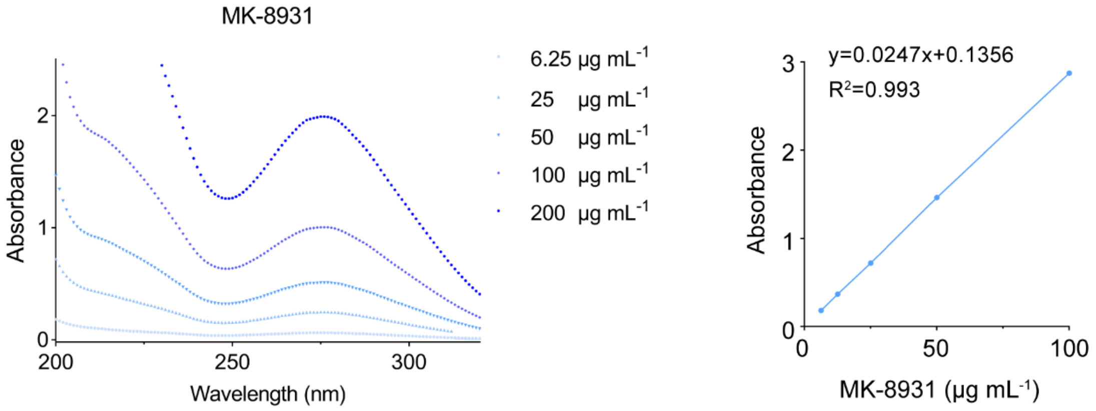

Figure S12. The standard curve of MK-8931 was calculated according to UV-Vis spectra.

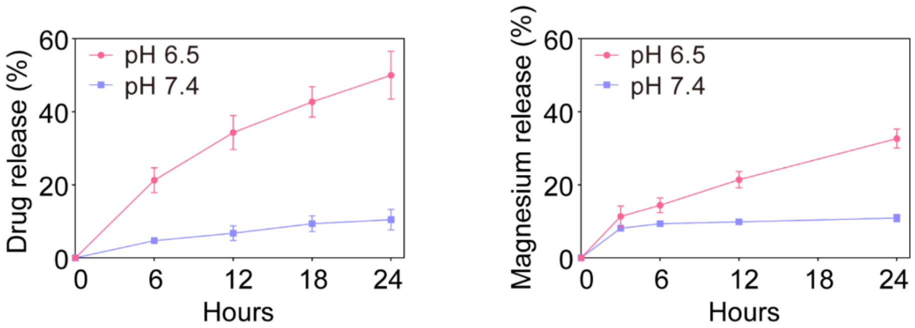

Figure S13. The pH-responsive release profiles of MK-8931 (replaced by model drug Nile red) and Mg^{2+} from Mg^{2+}@MK-8931@MPP in pH 7.4 and pH 6.5 solution.

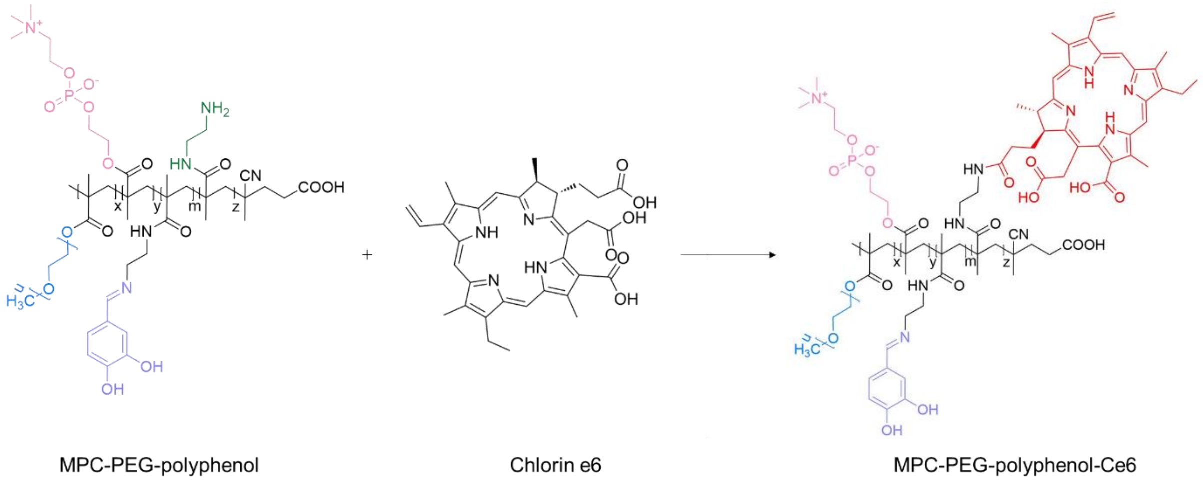

Figure S14. The synthesis route of Chlorin e6 (Ce6)-conjugated MPP (MPP-Ce6) polymer was synthesized to prepares prepare Ce6 labelled Mg^{2+}@MK-8931@MPP nanoparticles, naming Ce6@Mg^{2+}@MK-8931@MPP.

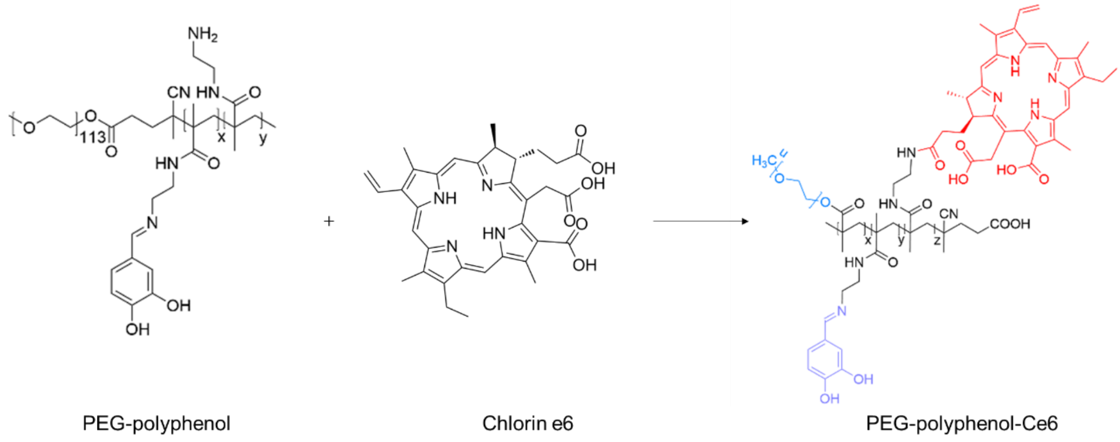

Figure S15. The synthesis route of Chlorin e6 (Ce6)-conjugated PP (PP-Ce6) polymer Structures of PEG-polyphenol (PP) and PP-IR780 were synthesized to prepare IR780Ce6 labelled Mg^{2+}@MK-8931@PP nanoparticles, naming IR780@Mg^{2+}@MK-8931@PP.

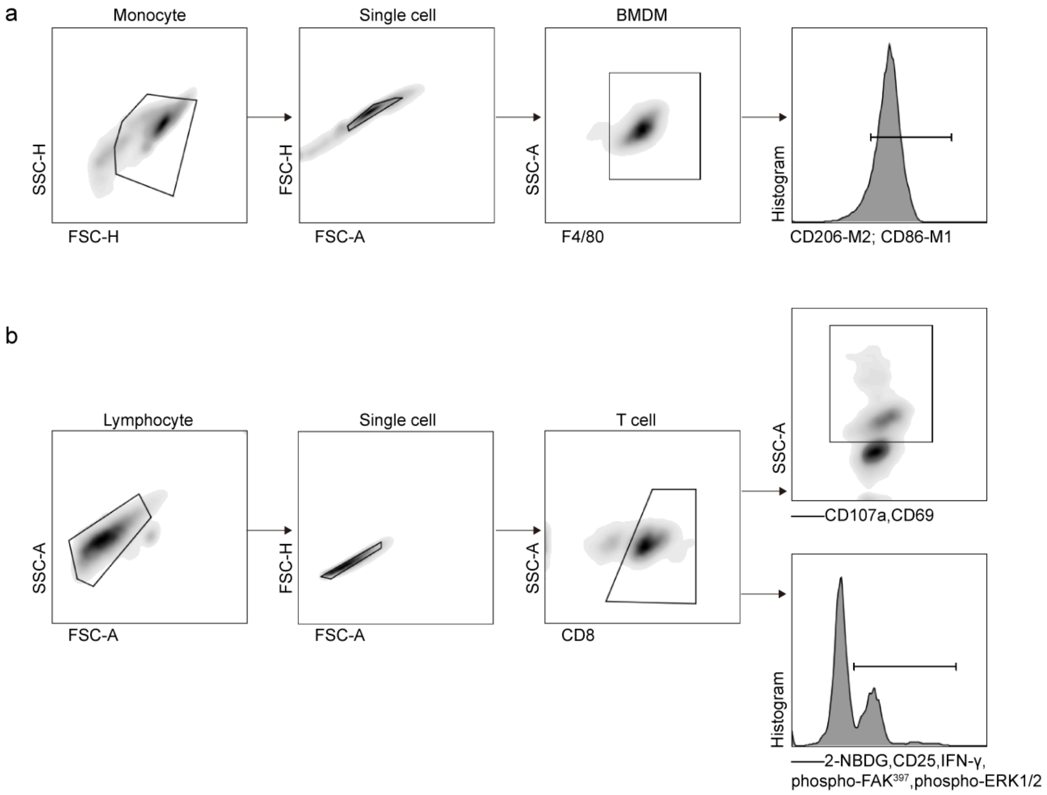

Figure S16. Gating strategy of in vitro flow cytometric analysis. a. M1-like BMDM ($CD86^+$ $F4/80^+$ gating on F4/80) and M2-like BMDM ($CD206^+$ $F4/80^+$ gating on F4/80) proportion analyses in vitro. b. Effector function analyses of CD8 T cells in vitro ($marker^+CD8a^+$ gating on CD8a). Since BMDM and CD8 T cells are specifically inducible or isolated, a simple staining strategy is sufficient, and since measurements can be made instantaneously, live/dead staining is not required.

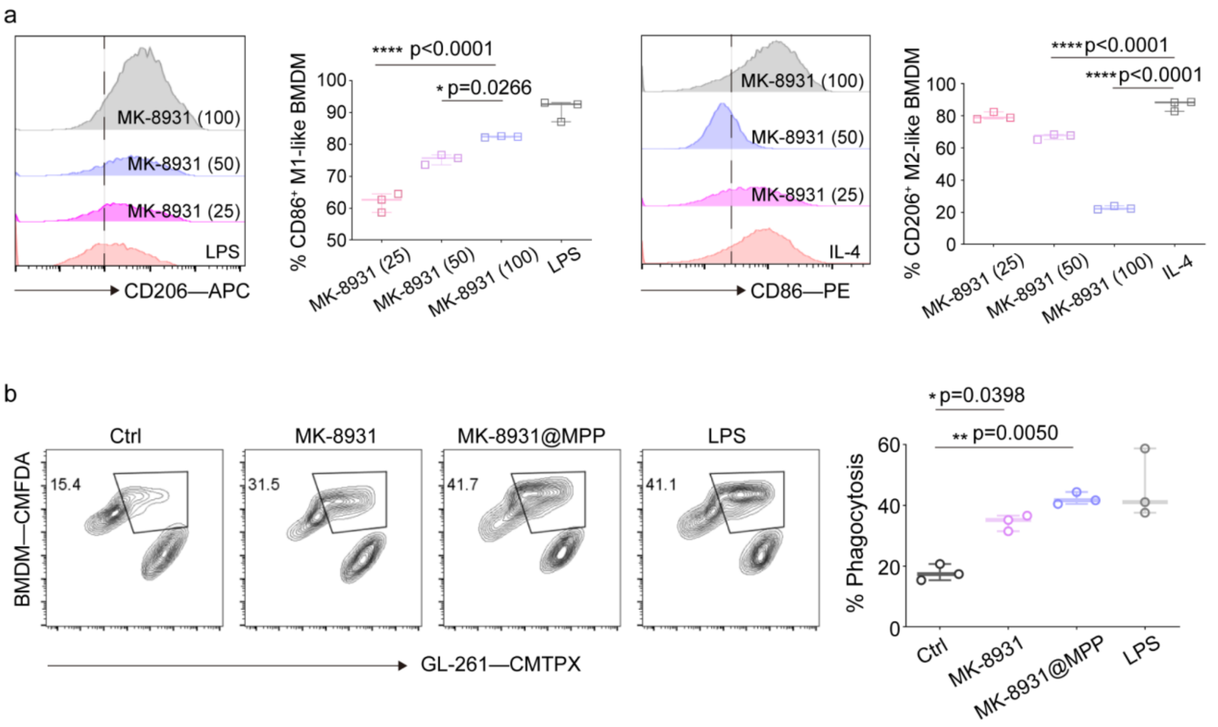

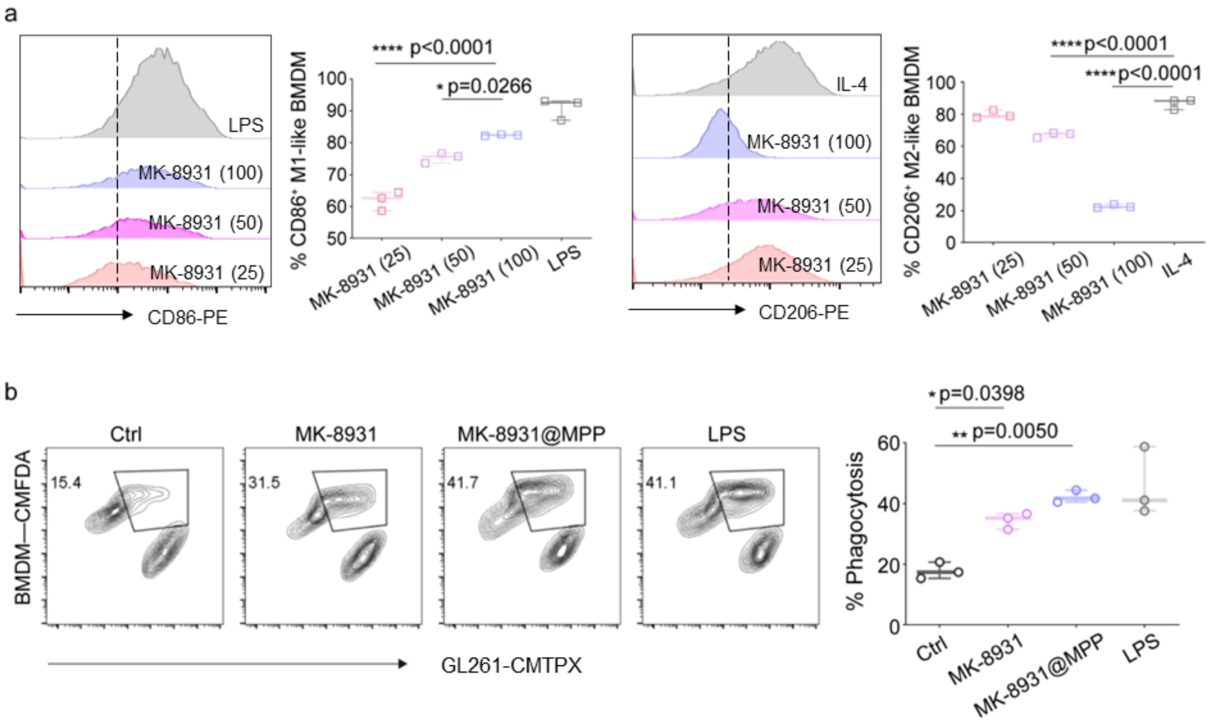

Figure S17. The representative flow cytometric plots and quantitative analyses of the CD206^{+} M2-like BMDMs, CD80^{+} M1-like BMDMs (a), and pretreated-BMDM-mediated GL261 phagocytosis (B) under the different treatments (MK-8931 concentration in each experiment, M1/M2-like detection: 25 μg mL^{-1}, 50 μg mL^{-1}, and 100 μg mL^{-1}; Phagocytosis: 50 μg mL^{-1}). n=3. Statistical significance was calculated via one-way ANOVA with Tukey's multiple comparisons.

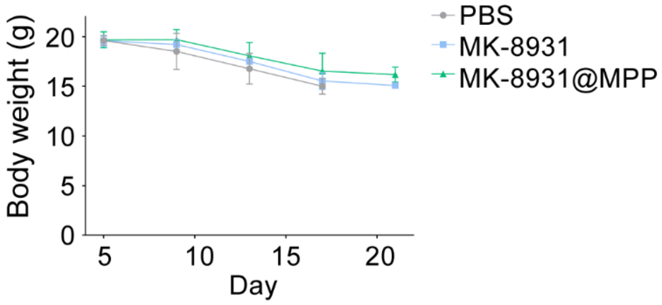

Figure S18. Body weight variation per group post Saline, MK-8931, and MK-8931@MPP treatments.

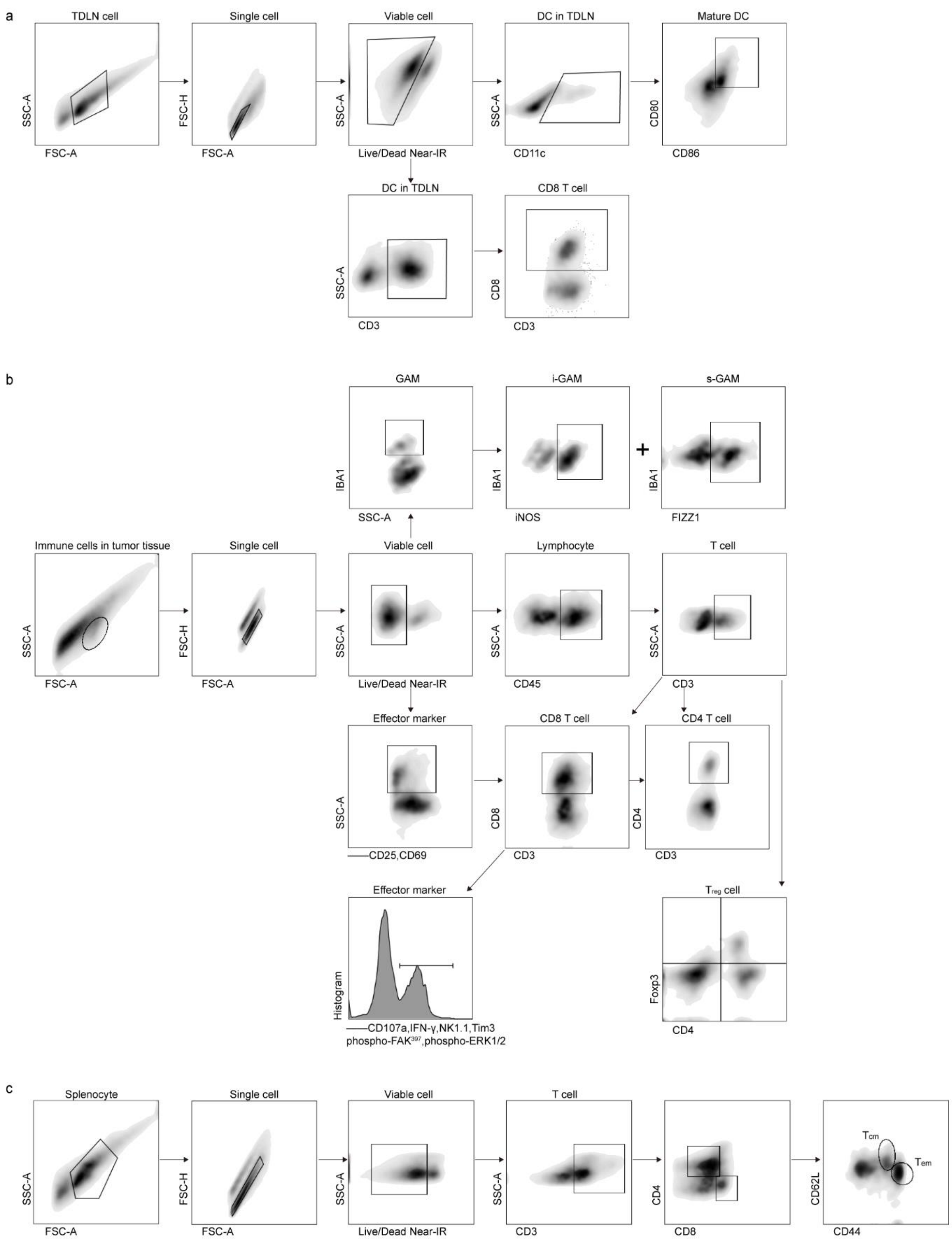

Figure S19. Gating strategy of in vivo flow cytometric analysis. a. Mature DCs (CD86$^+$CD80$^+$CD11c$^+$ gating on CD11c) and CD8 (CD8a$^+$CD3e$^+$ gating on CD3e) proportion analyses in tumor-draining lymph nodes (TDLNs) in vivo. b. Immune cell phenotype analyses in tumor tissues. i-GAM: iNOS$^+$IBA1$^+$ gating on IBA1; s-GAM: FIZZ1$^+$IBA1$^+$ gating on IBA1; CD8 T cell: CD8a$^+$CD3e$^+$CD45$^+$ gating on CD3e; effector marker: marker$^+$

$CD8a^+CD3e^+CD45^+$ gating on CD8a; CD4 T cell: $CD4^+CD3e^+CD45^+$ gating on CD3e; T_{reg} cell: $Foxp3^+CD4^+CD3e^+CD45^+$ gating on CD4 or CD3e. c. Effector memory (T_{em}, $CD62L^-CD44^+CD4^+/CD8a^+CD3e^+$ gating on CD4 or CD8a) and central memory (T_{cm}, $CD62L^+CD44^+CD4^+/CD8a^+CD3e^+$ gating on CD4 or CD8a) proportion analyses in the spleen.

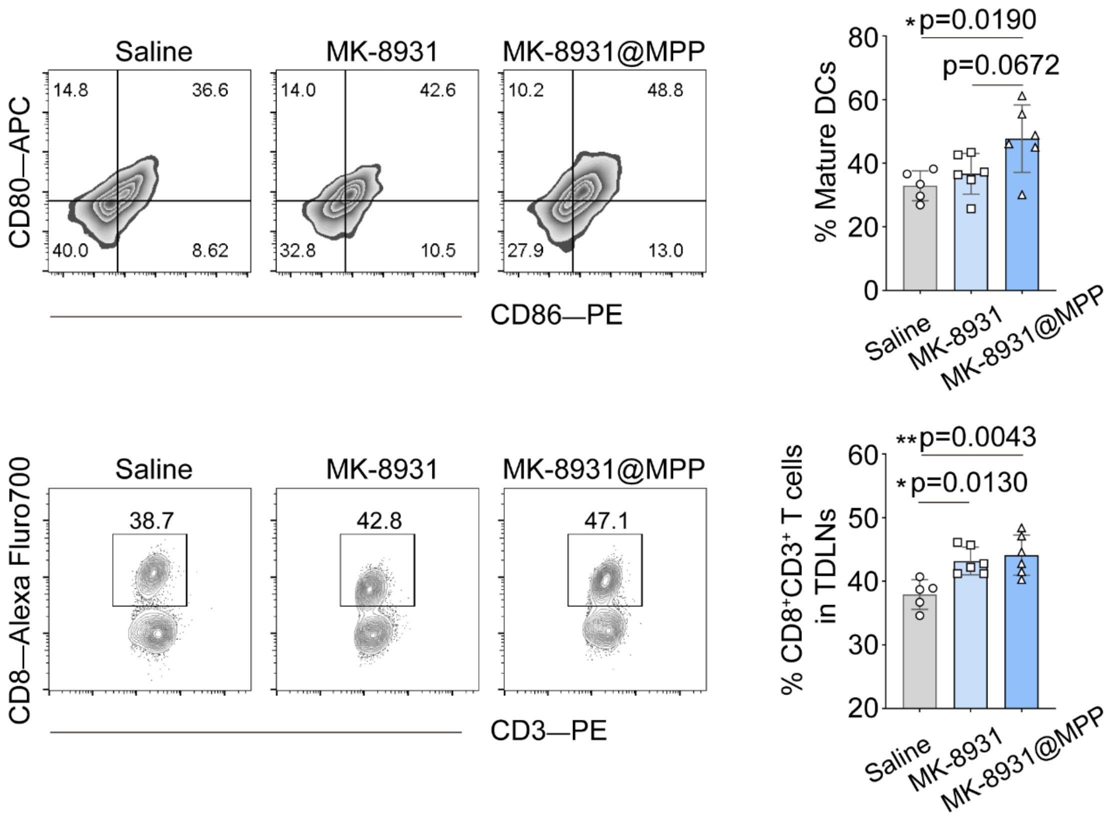

Figure S20. The representative flow cytometric plots and quantitative analysis of the maturation of dendritic cells and CD8 T cell proportion in tumor-draining lymph nodes (TDLNs). n=5 in Saline group, n=6 in MK-8931 and MK-8931@MPP groups. Statistical significance was calculated via one-way ANOVA with Tukey's multiple comparisons.

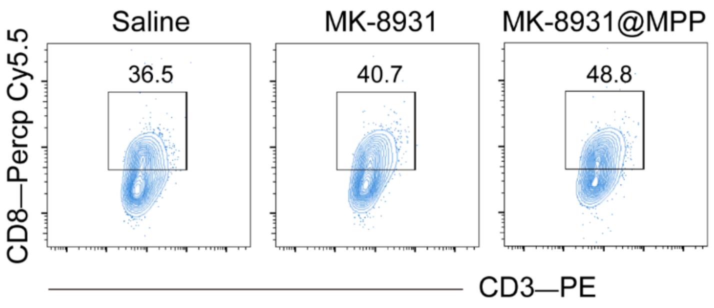

Figure S21. The representative flow cytometric plots ~~and quantitative analysis~~ of CD8$^+$CD3$^+$ T cells proportions in tumor tissues. ~~n=5 in saline group, n=6 in MK-8931 and MK-8931@MPP groups. Statistical significance was calculated via one-way ANOVA with Tukey's multiple comparisons.~~

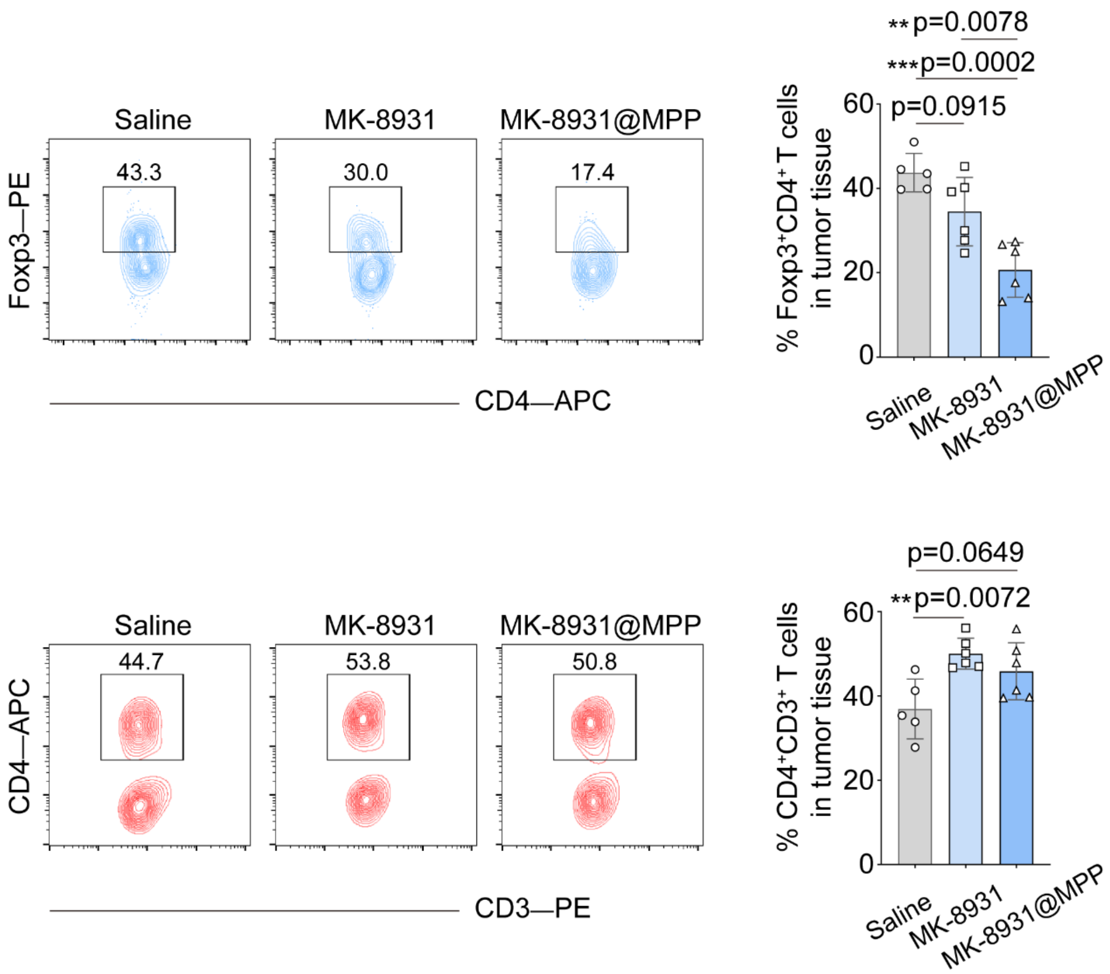

Figure S22. The representative flow cytometric plots and quantitative analysis of T_{reg} cells and $CD4^{+}CD3^{+}$ T cells proportions in tumor tissues. n=5 in Saline group, n=6 in MK-8931 and MK-8931@MPP groups. Statistical significance was calculated via one-way ANOVA with Tukey's multiple comparisons.

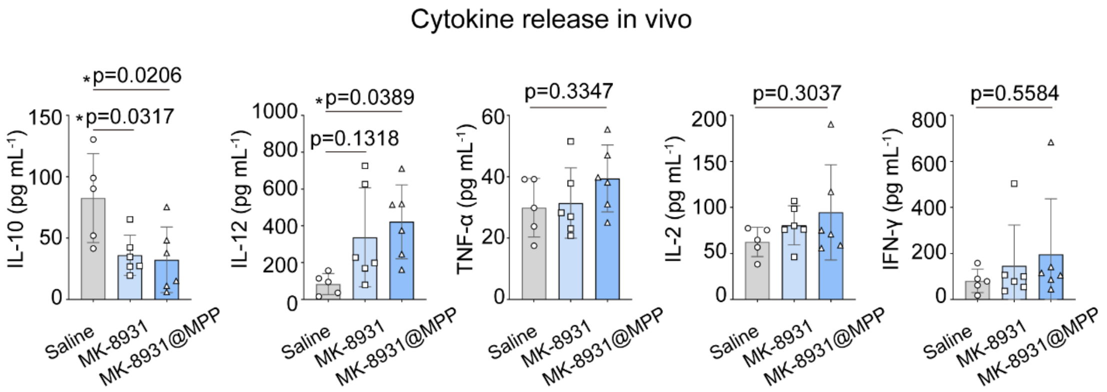

Figure S23. Enzyme-linked immunosorbent assay (ELISA) detection and quantitative analyses of cytokines in sera upon Saline, MK-8931, and MK-8931@MPP treatments. n=5 in Saline group, n=6 in MK-8931 and MK-8931@MPP groups. Statistical significance was calculated via one-way ANOVA with Tukey's multiple comparisons.

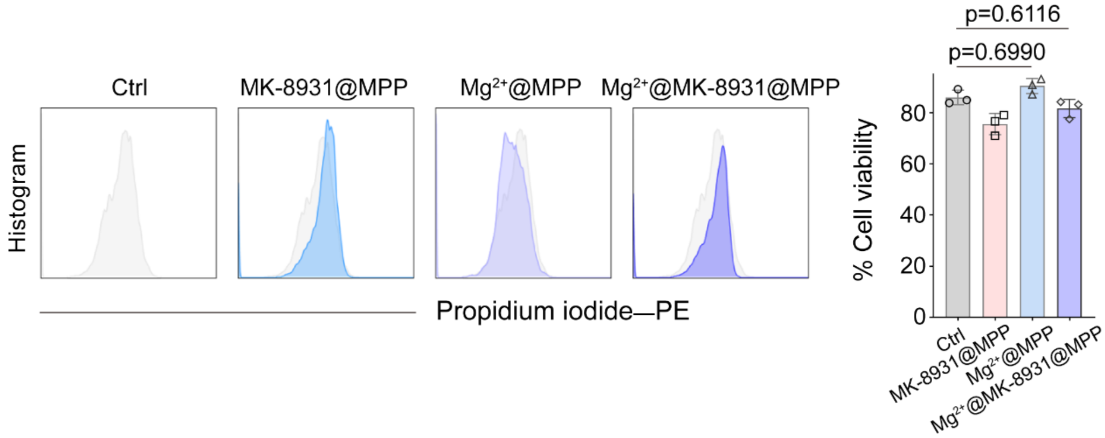

Figure S24. Toxicity of MK-8931@MPP, Mg^{2+}@MPP, and Mg^{2+}@MK-8931@MPP on CD8 T cells after 24 h co-incubation. n=3. Statistical significance was calculated via one-way ANOVA with Tukey's multiple comparisons.

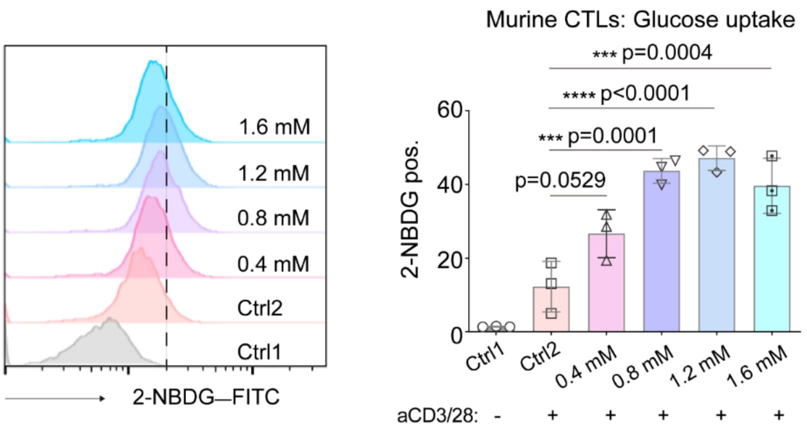

Figure S25. 2-NBDG detection for glucose uptake evaluation of CTLs induced by Mg^{2+} with 0, 0.4, 0.8, 1.2, and 1.6 mM concentrations.

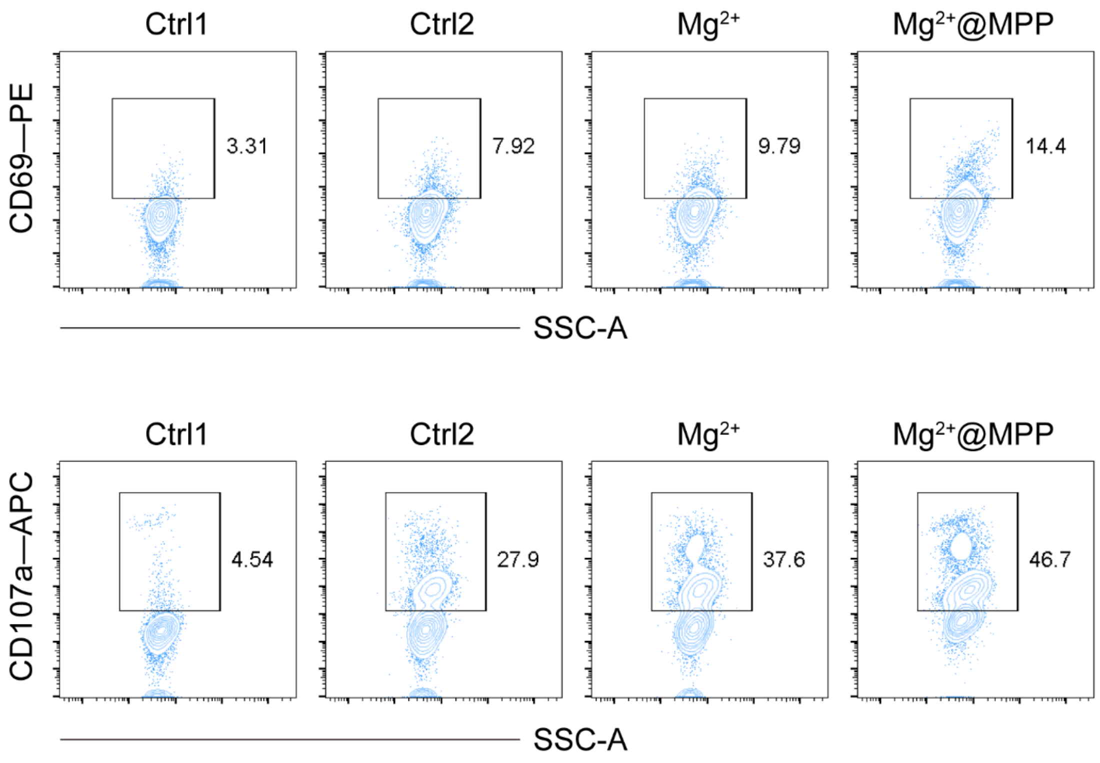

Figure S26. Representative flow cytometric plots of CD69 and CD107a after diverse treatments. Ctrl1 and Ctrl2 represent the CD8 T cells without and with aCD3/28 activation respectively; Both Mg^{2+} and Mg^{2+}@MPP function along with aCD3/28 activation.

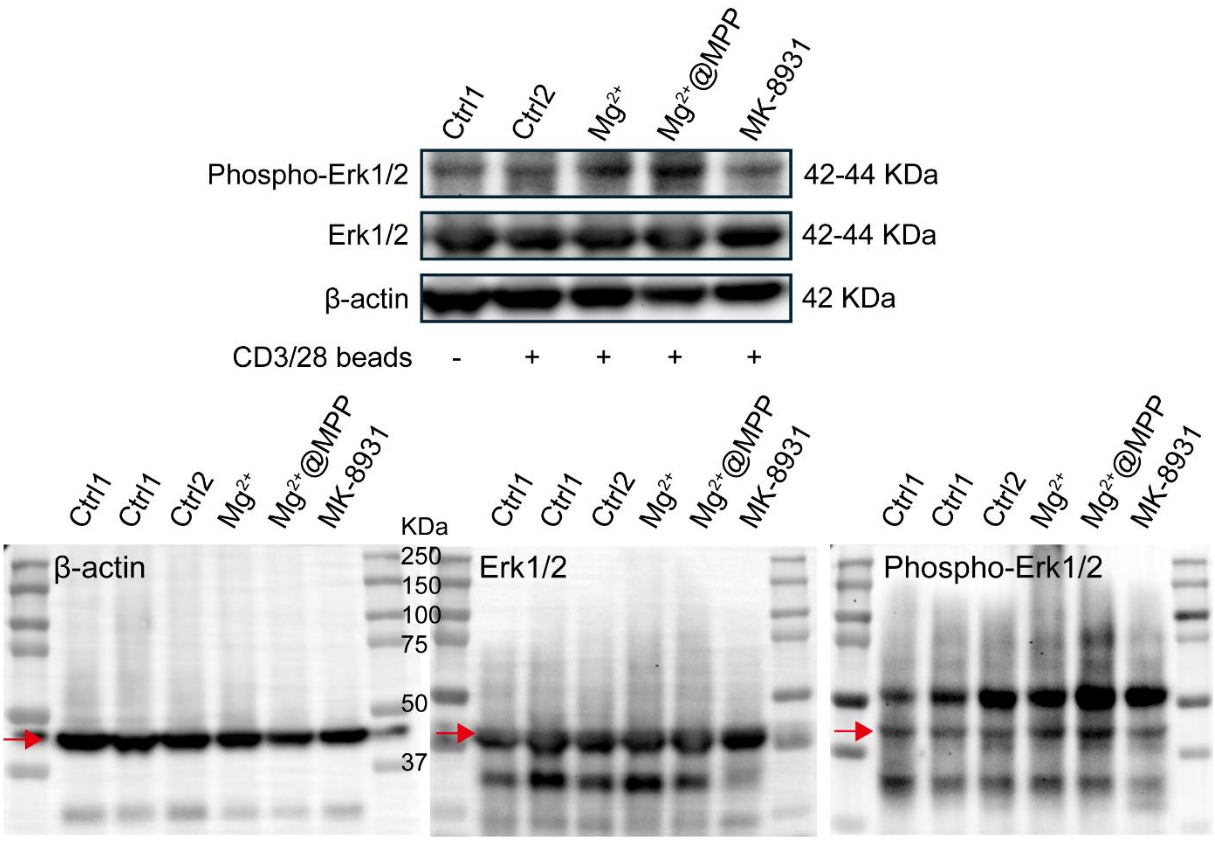

Figure S27. Western blotting results of Jurkat T cells with diverse treatments. The Jurkat cells were pretreated by human CD3/28 beads and followed by further treatments. Ctrl1 was defined as the naïve Jurkat T cells and Ctrl2 was the Jurkat cell with preactivation of human CD3/28 beads.

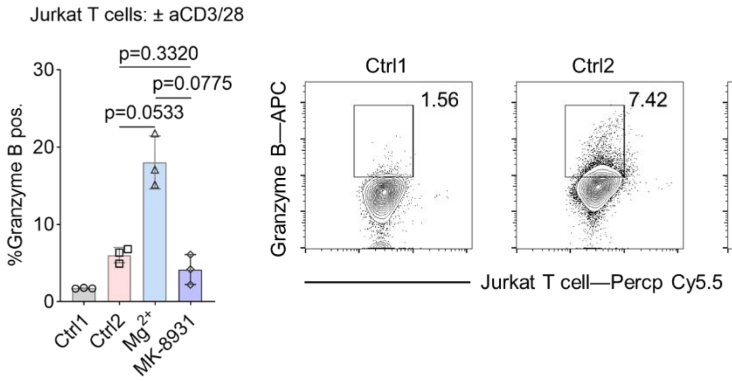

Figure S28. Quantitative analysis and representative flow cytometric plots of Jurkat T cells with diverse treatments. The Jurkat cells were pretreated by human CD3/28 beads and then treated with Mg^{2+} or MK-8931 treatments. Ctrl1 was defined as the naïve Jurkat T cells and Ctrl2 was the Jurkat cell with preactivation of human CD3/28 beads.

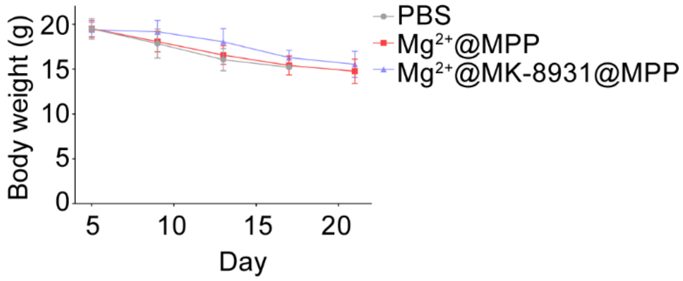

Figure S29. Body weight variation per group after Saline, Mg^{2+}@MPP, and Mg^{2+}@MK-8931@MPP treatments.

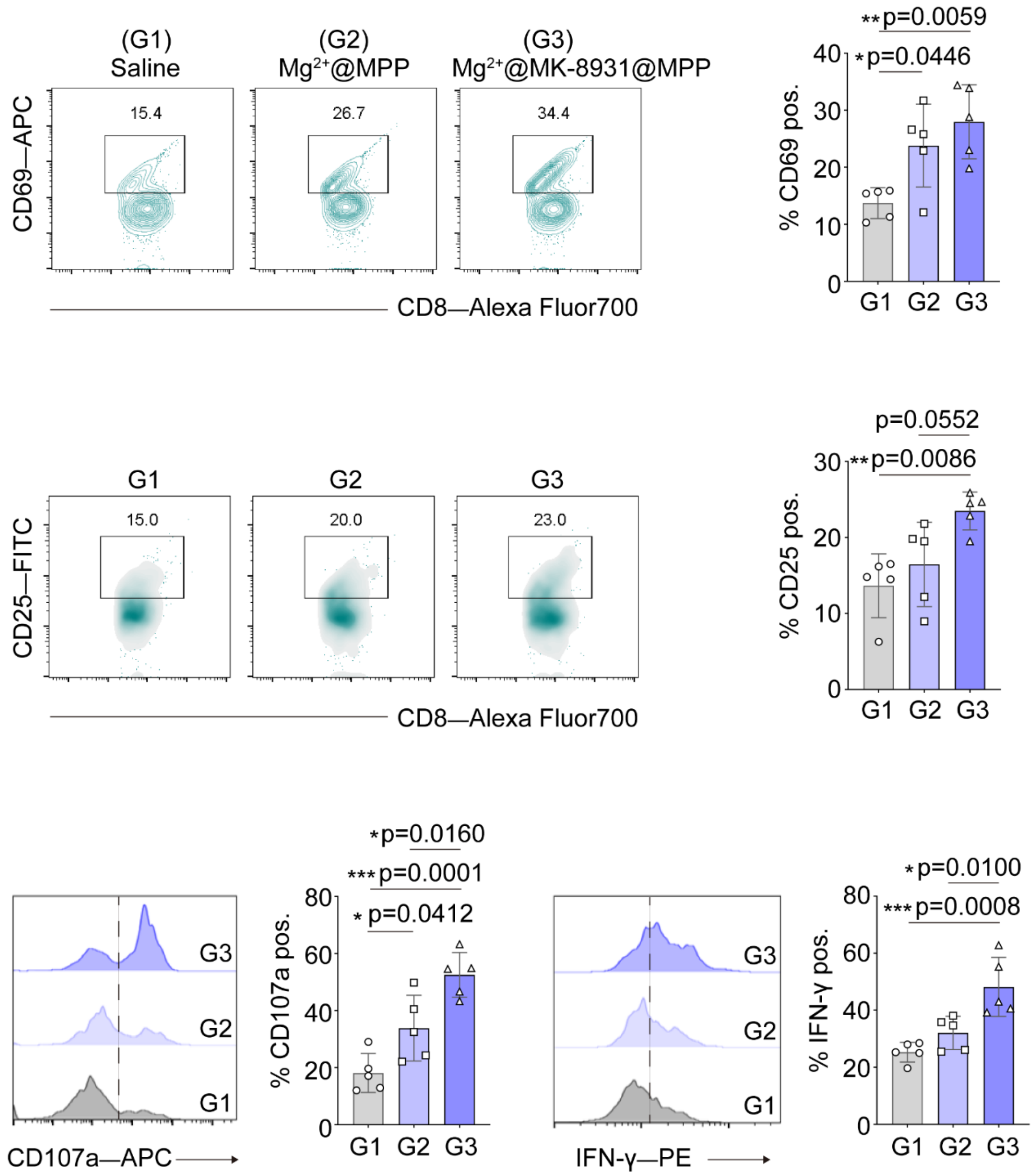

Figure S30. Proportional variation and flow cytometric quantification of CD8 T cell markers, including CD69 (early activation), CD25 (late activation), CD107a (degranulation), and IFN-γ (functional surrogate). n=5. Statistical significance was calculated via one-way ANOVA with Tukey's multiple comparisons.

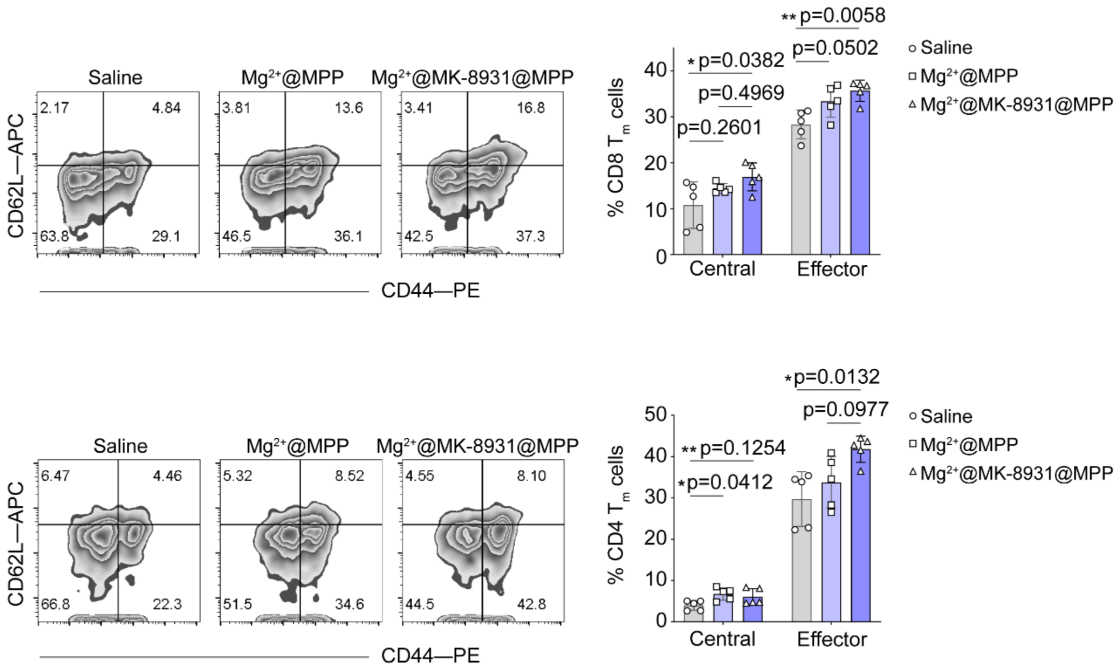

Figure S31. Proportional variation and flow cytometric quantification of effector memory (T_{em}, $CD62L^{-}CD44^{+}$) and central memory (T_{cm}, $CD62L^{+}CD44^{+}$) on CD4 T cells and CD8 T cells. n=5. Statistical significance was calculated via one-way ANOVA with Tukey's multiple comparisons.

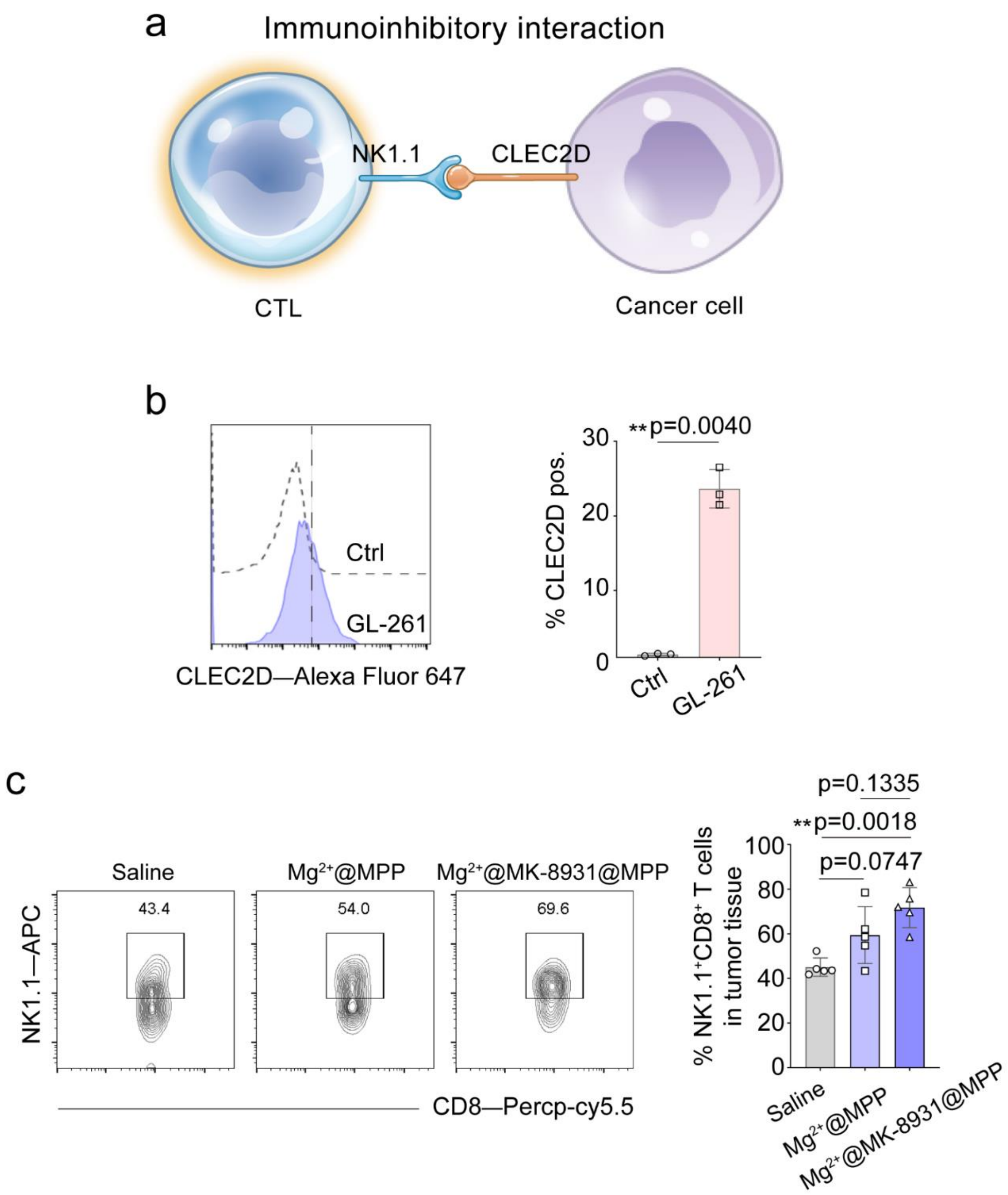

Figure S32. Verifying CLEC2D and NK1.1 expression on GL261 cancerous cells and glioblastoma-infiltrating CD8 T cells, respectively. a. Graphical illustration of CLEC2D-NK1.1 interaction-induced immune evasion. b. Representative histogram and quantification of CLEC2D expression on GL261 cancerous cells. c. Representative flow cytometric plots and quantification of NK1.1 expression on glioblastoma-infiltrating CD8 T cells. n=5. Statistical significance was calculated via one-way ANOVA with Tukey's multiple comparisons.

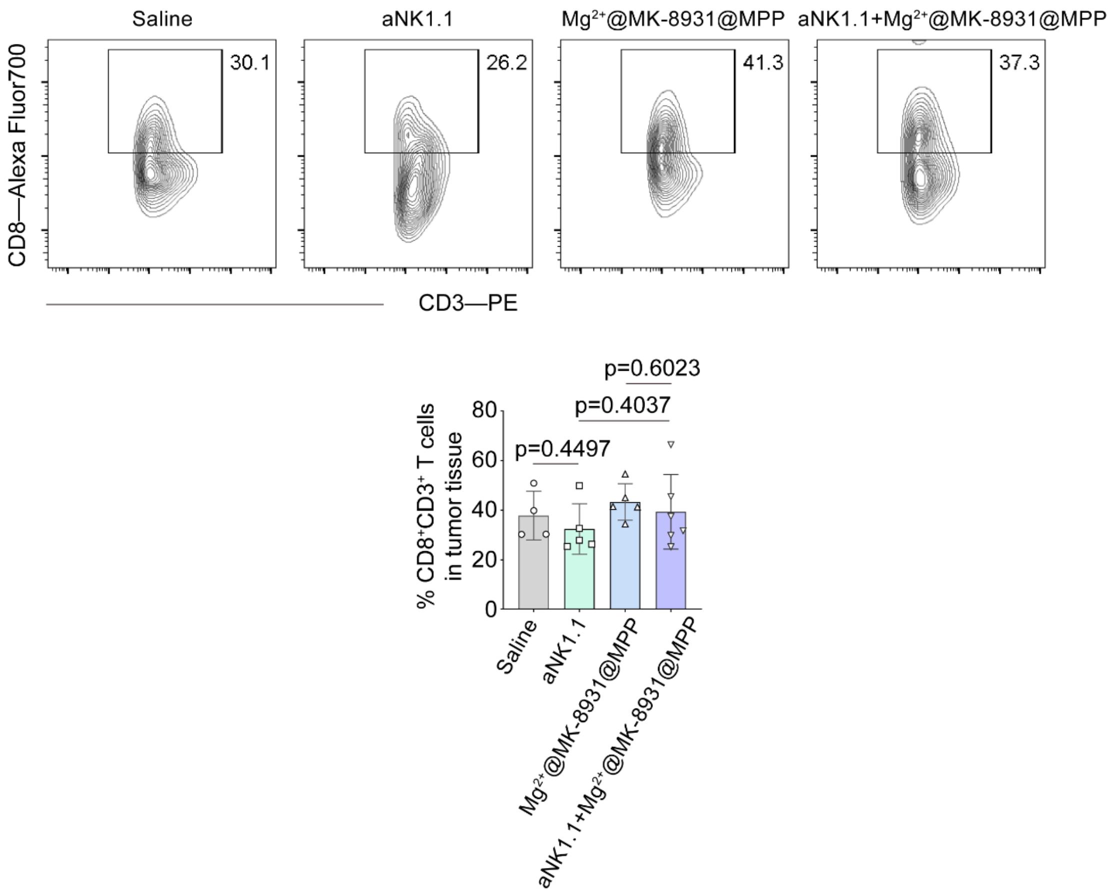

Figure S33. Representative flow cytometric plots and quantification of $CD8^+$ T cells ($CD8a^+CD3e^+CD45^+$ T cells gating on CD3e) in tumor tissue under Saline, aNK1.1, Mg^{2+}@MK-8931@MPP, and aNK1.1+Mg^{2+}@MK-8931@MPP treatments. Statistical significance was calculated via an unpaired two-tailed *t*-test with two-group comparisons.

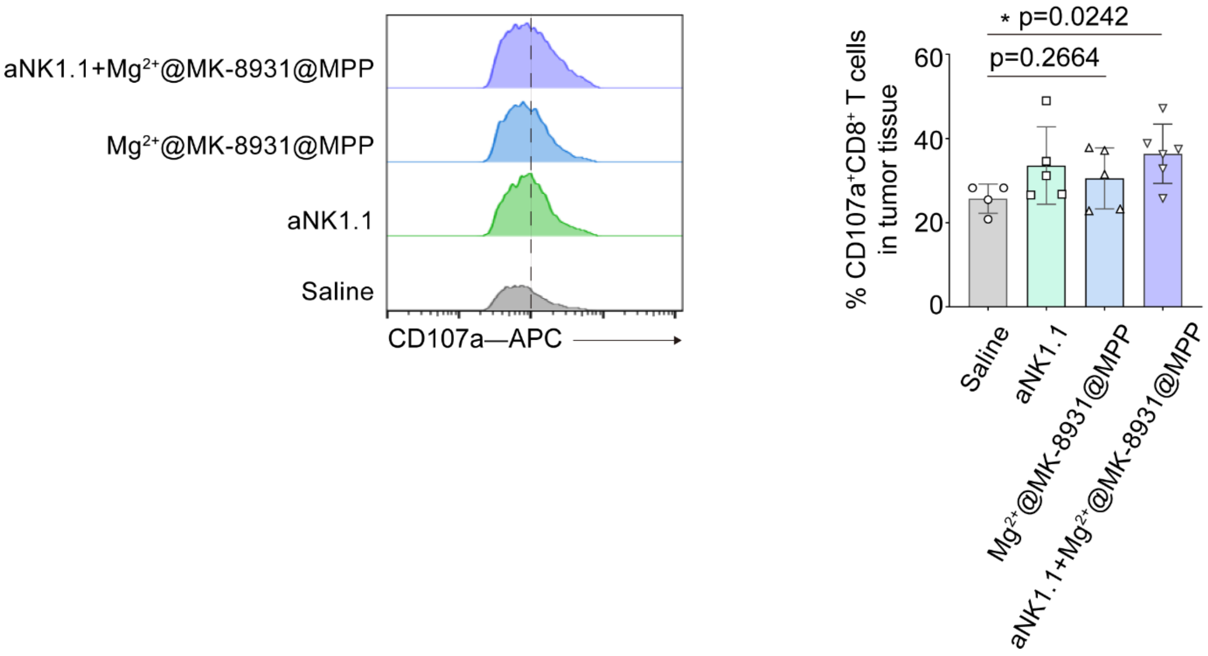

Figure S34. Representative histogram and quantitative analyses of CD107a expression on glioblastoma-infiltrating CD8 T cells ($CD107a^+CD8a^+CD3e^+CD45^+$ T cells gating on CD8a) under Saline, aNK1.1, Mg^{2+}@MK-8931@MPP, and aNK1.1+Mg^{2+}@MK-8931@MPP treatments. Statistical significance was calculated via an unpaired two-tailed *t*-test with two-group comparisons.

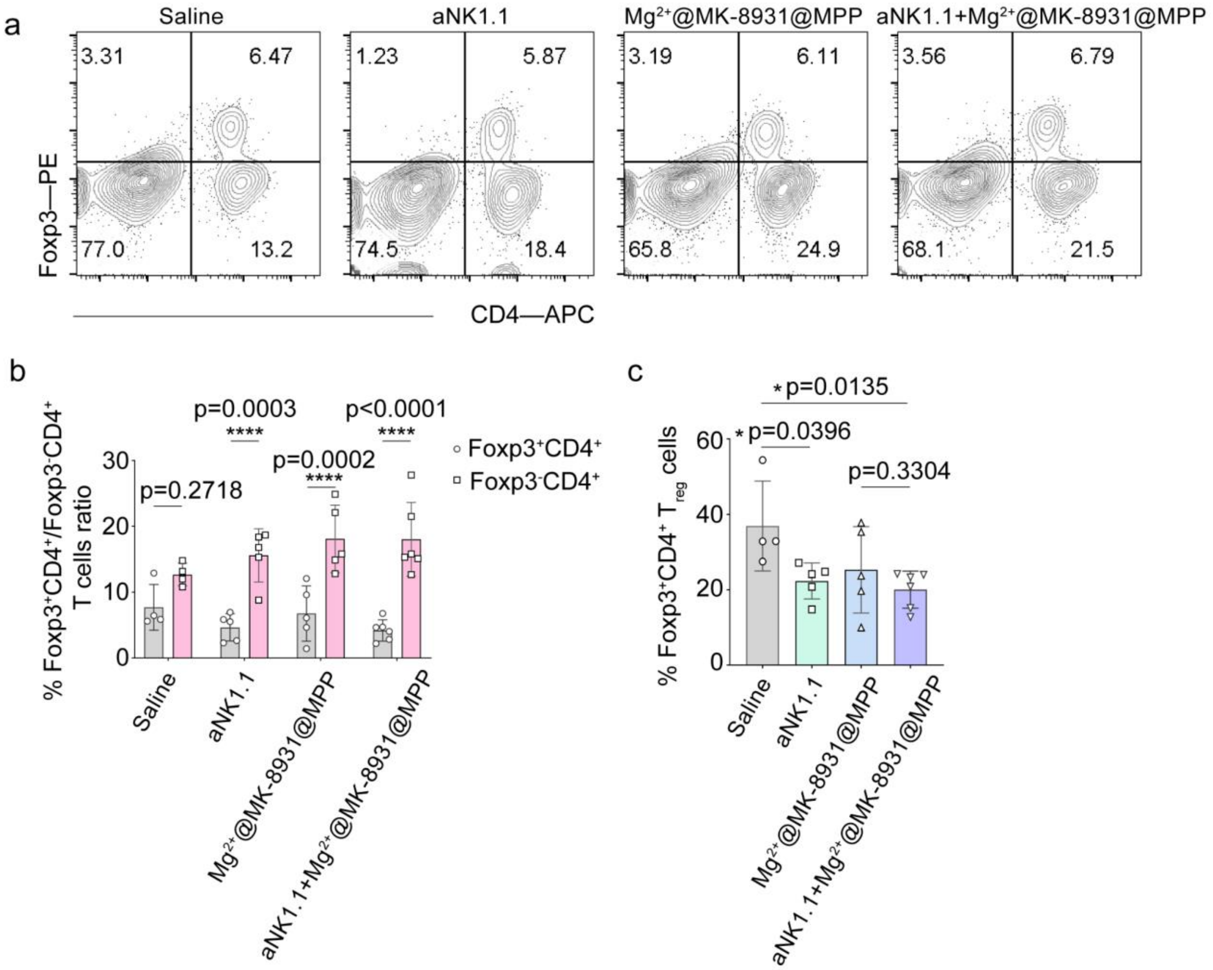

Figure S35. a,b. Representative flow cytometric plots and quantitative analyses of Foxp3$^+$ CD4$^+$/Foxp3$^-$CD4$^+$ T cells gating on CD3. c. Quantitative analysis of Foxp3$^+$CD4$^+$ T$_{reg}$ cells gating on CD4. Statistical significance was calculated via two-way ANOVA with Sidak's multiple comparisons test for (**b**). Statistical significance was calculated via unpaired two-tailed *t*-test with two-group comparisons for (**c**).

Supplementary Table 1. Chemical reagents and functional kits

Reagent	Cat #	Source
**Magnesium nitrate hexahydrate**	13446-18-9	Sigma-Aldrich
**Verubecestat (MK-8931)**	DC9901	DC Chemicals
**Nile red**	7385-67-3	MedChemExpress
**2,2,2-Tribromoethanol**	75-80-9	Sigma-Aldrich
**2-Methyl-2-butanol**	75-85-4	Sigma-Aldrich

**Nitric acid**	7697-37-2	Sigma-Aldrich
**Hydrochloric acid**	7647-01-0	Sigma-Aldrich
**Sodium Pyruvate**	P4562-25g	Sigma-Aldrich
**Glucose**	50-99-7	Sigma-Aldrich
**Oligomycin**	1404-19-9	Sigma-Aldrich
**2-deoxy-glucose**	29702-43-0	Sigma-Aldrich
**Phosphate-buffered saline**	70011044	Thermo Fisher Scientific
**HEPES**	15630080	Thermo Fisher Scientific
**Propidium Iodide**	P1304MP	Thermo Fisher Scientific
**SYTOX™ Green Nucleic Acid Stain**	S7020	Invitrogen
**CellTracker™ Red CMTPX Dye**	C34552	Invitrogen
**Antifade Mounting Medium with DAPI**	P0131-25ml	Beyotime
**Lipopolysaccharide (LPS)**	00-4976-93	eBioscience
**ACK Lysing Buffer**	A1049201	Thermo Fisher Scientific
**β-Mercaptoethanol**	M3148	Sigma-Aldrich
**L-Glutathione reduced**	G6013-10G	Sigma-Aldrich
**Phorbol 12-myristate 13-acetate (PMA)**	P849986-1mg	Macklin
**Ionomycin**	I838446-1mg	Macklin
**Poly-D-lysine**	E607014	Sangon Biotech
**VivoGlo™ Luciferin**	P1043	Sigma-Aldrich
**Collagenase type III**	LS0004182	Worthington
**Hyaluronidase**	H3506	Sigma-Aldrich
**DNase I**	58C10349	Worthington
**Slide-A-Lyzer MINI dialysis device (3.5K MWCO)**	88400	Thermo Fisher Scientific
**Poly (ethylene glycol) methyl ether methacrylate**	26915-72-0	Aladdin

**2-Methacryloyloxyethyl phosphorylcholine**	67881-98-5	Aladdin
**4-Cyano-4-(phenylcarbonothioylthio) pentanoic Acid**	201611-92-9	Aladdin
**2,2'-Azobis(2-methylpropionitrile**	78-67-1	Aladdin
**Trifluoroacetic acid**	76-05-1	Aladdin
**3,4-Dihydroxybenzaldehyde**	139-85-5	Aladdin
**IR-780 iodide**	207399-07-3	Aladdin
**2-NBDG**	ab287845	Abcam
**Mouse IL-2 ELISA kit**	LEM020-2	Laizee
**Mouse IL-10 ELISA kit**	EMC005.95.2	NeoBioscience
**Mouse TNF-α ELISA kit**	EMC102a.96.2	NeoBioscience
**Mouse IL-12p70 ELISA kit**	EMC006.96.2	NeoBioscience
**Mouse IFN-γ ELISA kit**	EMC101g.96.2	NeoBioscience
**EasySep™ Mouse CD8^{+} T cell isolation kit**	19853	Stemcell
**CytoTox 96® non-radioactive cytotoxicity assay kit**	G1780	Promega
**Dynabeads™ Human T-Activator CD3/CD28 for T Cell Expansion and Activation**	11131D	Invitrogen
**LIVE/DEAD™ fixable near-IR dead cell stain kit**	L10119	Invitrogen
**BUN assay kit**	C013-2-1	NanJing JianCheng Bioengineering Institute
**CRE assay kit**	C011-2-1	NanJing JianCheng Bioengineering Institute
**AST assay kit**	C010-2-1	NanJing JianCheng Bioengineering Institute

**ALT assay kit**	C009-2-1	NanJing JianCheng Bioengineering Institute

Supplementary Table 2. Recombinant proteins and antibodies

**Antibodies or Cytokines**	**Cat #**	**Source**
**Anti-Mouse NK1.1 – Purified in vivo PLATINUM™ Functional Grade (Clone PK136)**	N268	Leinco
**IBA1 Polyclonal Antibody**	PA5-27436	Invitrogen
**p44/42 MAPK (Erk1/2) (137F5) Rabbit mAb**	4695S	Cell signaling Technology
**Phospho p44/42 MAPK ERK1/2 (Thr202 Tyr204)**	4370L	Cell signaling Technology
**β-Actin Antibody**	4967S	Cell signaling Technology
**Phospho-FAK (Tyr397) Recombinant Rabbit Monoclonal Antibody (Clone 31H5L17)**	700255	Invitrogen
**Recombinant Anti-iNOS antibody**	ab178945	Abcam
**TruStain FcX™ PLUS (anti-mouse CD16/32) Antibody (Clone S17011E)**	156604	BioLegend
**Animal-Free Recombinant Murine M-CSF**	AF-315-02	Peprotech
**Animal-Free Recombinant Murine GM-CSF**	AF-315-03-50	Peprotech
**Recombinant Murine IL-4**	214-14	Peprotech
**Recombinant Murine IL-2**	212-12	Peprotech
**Purified Hamster Anti-Mouse CD28**	553295	BD Pharmingen
**Purified anti-mouse CD3ε Antibody (Clone 145-2C11)**	100340	BioLegend

**CD45 Monoclonal Antibody, FITC, PE, Percp-cy5.5**	11-0451-82; 12-0451-83; 45-0451-82	eBioscience
**CD3e Monoclonal Antibody, FITC, PE, Percp-cy5.5**	11-0031-82; 12-0031-83; 45-0031-82	eBioscience
**CD4 Monoclonal Antibody, FITC, PE, APC**	11-0041-82; 17-0041-83	eBioscience
**Anti-mouse CD4 Antibody, PE**	100408	BioLegend
**CD8a Monoclonal Antibody, Percp cy5.5, APC, Alexa Fluor 700**	45-0081-82; 17-0081-83; 56-0081-82	eBioscience
**Anti-mouse FOXP3 Antibody, PE (Clone MF-14)**	126404	BioLegend
**CD80 (B7-1) Monoclonal Antibody (16-10A1), APC**	17-0801-82	eBioscience
**CD86 (B7-2) Monoclonal Antibody (GL1), PE**	12-0862-82	eBioscience
**F4/80 Monoclonal Antibody (BM8), PE-Cyanine5**	15-4801-82	eBioscience
**CD206 (MMR) Monoclonal Antibody (MR6F3), PE**	12-2061-82	eBioscience
**CD11c Monoclonal Antibody (N418), FITC**	11-0114-85	eBioscience
**CD11b Monoclonal Antibody (ICRF44), FITC**	11-0118-42	eBioscience
**Anti-mouse CD366 (Tim-3) Antibody, Percp-cy5.5 (Clone RMT3-23)**	119718	BioLegend
**CD44 Monoclonal Antibody (IM7), PE**	12-0441-81	eBioscience
**CD62L (L-Selectin) Monoclonal Antibody (MEL-14), APC**	17-0621-83	eBioscience

**CD206 (MMR) Monoclonal Antibody (MR6F3), PE**	12-2061-82	eBioscience
**Anti-human/mouse Granzyme B Recombinant Antibody, APC**	372204	BioLegend
**IFN gamma Monoclonal Antibody (XMG1.2), PE**	12-7311-82	eBioscience
**TNF alpha Monoclonal Antibody (MP6-XT22), FITC**	11-7321-82	eBioscience
**iNOS Monoclonal Antibody (Clone CXNFT), PE**	12-5920-82	eBioscience
**RELM alpha Monoclonal Antibody (Clone DS8RELM), PerCP-eFluor™ 710**	46-5441-82	eBioscience
**Anti-mouse CD107a (LAMP-1) Antibody, APC**	121614	BioLegend
**Anti-mouse CD25 Antibody, APC**	101910	BioLegend
**Anti-mouse CD69 Antibody, APC**	104514	BioLegend
**Anti-mouse NK-1.1 Antibody, APC**	108710	BioLegend
**OCIL/CLEC2d Antibody (Clone 166C1309), Alexa Fluor® 647**	NB100-56553AF647	Novus biologicals
**Anti-Iba1 Antibody (Clone 1022-5), FITC**	sc-3272	Santa Cruz
**Phospho-FAK (Tyr397)**	700255	Invitrogen
**Anti-ERK1/2 Phospho (Thr202/Tyr204) Antibody, Alexa Fluor 647**	369504	BioLegend
**Goat anti-Rabbit IgG (H+L) Highly Cross-Adsorbed Secondary Antibody, Alexa Fluor 647**	A-21245	Invitrogen
**Goat anti-Mouse IgG (H+L) Cross-Adsorbed Secondary Antibody, Alexa Fluor 488**	A-11001	Invitrogen

Supplementary Table 3. Organisms/strains, cell lines, and products for cell culture

Product name	Cat #	Supplier
**GL261 cell line**	ZQ0932	Shanghai Zhong Qiao Xin Zhou Biotechnology Co, Ltd
**GL261-luc cell line**	LZQ0065	Shanghai Zhong Qiao Xin Zhou Biotechnology Co, Ltd
**b.End3 cell line**		Provided by Dr. Lisi Xie, Sun Yat-Sen University
**RAW264.7 cell line**	TIB-71	The American Type Culture Collection
**Jurkat T cell line**	Clone E6-1	Noblebio
**Roswell Park Memorial Institute 1640 Medium**	11875093	Gibco
**Roswell Park Memorial Institute 1640 Medium, no glucose**	11879020	Gibco
**Dulbecco's Modified Eagle Medium**	11965092	Gibco
**Dulbecco's Modified Eagle Medium, no glucose**	11966025	Gibco
**Fetal bovine serum**	26140079	Gibco
**Penicillin-streptomycin**	15140122	Gibco
**C57BL/6J mice**		Animal Facility University of Macau
**Rag1$^{-/-}$ C57BL/6J mice**		Animal Facility University of Macau
